# A Group-Facilitated, Internet-Based Intervention to Promote Mental Health and Well-Being in a Vulnerable Population of University Students: Randomized Controlled Trial of the Be Well Plan Program

**DOI:** 10.2196/37292

**Published:** 2022-05-05

**Authors:** Daniel B Fassnacht, Kathina Ali, Joep van Agteren, Matthew Iasiello, Teri Mavrangelos, Gareth Furber, Michael Kyrios

**Affiliations:** 1 College of Education, Psychology and Social Work Flinders University Adelaide Australia; 2 Órama Institute for Mental Health and Wellbeing Flinders University Adelaide Australia; 3 Wellbeing and Resilience Centre South Australian Health and Medical Research Institute Adelaide Australia; 4 College of Nursing and Health Science Flinders University Adelaide Australia; 5 Health, Counselling & Disability Services Flinders University Adelaide Australia

**Keywords:** COVID-19, mental health, well-being, depression, anxiety, online, digital, intervention, Be Well Plan, health outcome, online health, digital health, health intervention, primary outcome, cognition, randomized control trial, resilience, participant satisfaction, student

## Abstract

**Background:**

A growing literature supports the use of internet-based interventions to improve mental health outcomes. However, most programs target specific symptoms or participant groups and are not tailored to facilitate improvements in mental health and well-being or do not allow for needs and preferences of individual participants. The Be Well Plan, a 5-week group-facilitated, internet-based mental health and well-being group intervention addresses these gaps, allowing participants to select a range of activities that they can tailor to their specific characteristics, needs, and preferences.

**Objective:**

This study aims to test whether the Be Well Plan program was effective in improving primary outcomes of mental well-being, resilience, anxiety, and depression compared to a waitlist control group during the COVID-19 pandemic; secondary outcomes included self-efficacy, a sense of control, and cognitive flexibility. The study further seeks to examine participants’ engagement and satisfaction with the program.

**Methods:**

A randomized controlled trial (RCT) was conducted with 2 parallel arms, an intervention and a waitlist control group. The intervention involved 5 weekly 2-hour sessions, which were facilitated in group format using Zoom videoconferencing software. University students were recruited via social media posts, lectures, emails, flyers, and posters.

**Results:**

Using an intentional randomization 2:1 allocation strategy, we recruited 215 participants to the trial (n=126, 58.6%, intervention group; n=89, 41.4%, waitlist control group). Of the 126 participants assigned to the intervention group, 75 (59.5%) commenced the program and were included in modified intention-to-treat (mITT) analyses. mITT intervention participants attended, on average, 3.41 sessions (SD 1.56, median 4); 55 (73.3%) attended at least 4 sessions, and 25 (33.3%) attended all 5 sessions. Of the 49 intervention group participants who completed the postintervention assessment, 47 (95.9%) were either very satisfied (n=31, 66%) or satisfied (n=16, 34%). The mITT analysis for well-being (*F*_1,162_=9.65, *P*=.002, Cohen *d*=0.48) and resilience (*F*_1,162_=7.85, *P*=.006, Cohen *d*=0.44) showed significant time × group interaction effects, suggesting that both groups improved over time, but the Be Well Plan (intervention) group showed significantly greater improvement compared to the waitlist control group. A similar pattern of results was observed for depression and anxiety (Cohen *d*=0.32 and 0.37, respectively), as well as the secondary outcomes (self-efficacy, Cohen *d*=0.50; sense of control, Cohen *d*=0.42; cognitive flexibility, Cohen *d*=0.65). Larger effect sizes were observed in the completer analyses. Reliable change analysis showed that the majority of mITT participants (58/75, 77.3%) demonstrated a significant reliable improvement in at least 1 of the primary outcomes.

**Conclusions:**

The Be Well Plan program was effective in improving mental health and well-being, including mental well-being, resilience, depression, and anxiety. Participant satisfaction scores and attendance indicated a high degree of engagement and satisfaction with the program.

**Trial Registration:**

Australian New Zealand Clinical Trial Registry ACTRN12621000180819; https://tinyurl.com/2p8da5sk

## Introduction

### Background

Despite increased investment and a growing awareness and acceptance of the need to address mental illness in an evidence-based way, to date, the prevalence of mental illness worldwide has not reduced [1,2]. On the contrary, the burden of mental illness on society is expected to grow in the next decade, both in economic and in health terms [3,4]. In addition to the significant proportion of individuals experiencing a diagnosable mental illness [5], many individuals experience poor mental health without a diagnosis (ie, psychological distress or low mental well-being), often referred to as the languishing group [6]. Despite suboptimal mental health (ie, the total number of individuals experiencing a mental illness, psychological distress, or low mental well-being) affecting a large proportion of the population, our health care systems and associated expenditures are devoted to servicing a small proportion of individuals with the most severe mental illnesses in tertiary or community care [7]. With respect to the use of evidence-based psychological therapies, as is seen in many mental health systems globally (eg, the National Health System in the United Kingdom and the Better Access initiative through the Medical Benefits Schedule in Australia), these are predominantly focused on providing care for clients with comparatively complex problems, such as moderate-to-severe common mental illnesses. Access to evidence-based, widely accessible help for less severe mental health needs is limited, ultimately leaving a large group at risk to develop more serious problems, particularly during times of community stress, such as global pandemics, which in turn is likely to increase the incidence of mental disorders. Therefore, a focus on prevention and early intervention should be a priority if we realistically wish to reduce the growing burden of suboptimal mental health [8].

### Stepped Care Models in Mental Health Promotion

Stepped care models have been proposed and implemented as a solution to resourcing challenges and subsequent access issues across the spectrum of mental health care [9,10]. These models aim to improve the match of service needs to symptom severity and complexity, while ensuring similar or improved effectiveness compared to care as usual [11,12]. The aim is to ensure that highly specialized care will mainly focus on more severe cases, while ensuring that appropriate and effective evidence-based help remains available for those with less severe needs. A well-known example of a system using stepped care principles is the United Kingdom’s Improving Access to Psychological Therapies (IAPT) system [13], where individuals have access to psychological interventions according to their needs (eg, offering low-cost and low-intensity guided self-help or group-based services as an initial step). Other examples of stepped care models can be readily found in the Western world, including in European countries and Australia [14,15].

Although stepped models of care theoretically include a focus on building or promoting good *mental health*, the models are conceptually designed to deal with the impact of *illness*. As such, the solutions implemented across the continuum focus on preventing or treating *symptoms of illness*, not necessarily promoting *good mental health* [16,17]. Although this differentiation seems semantic at first glance, when considering a parallel with physical health, clear differences can and should be noted. The treatment of symptoms of physical illness and activities to promote general physical health (or fitness) do not necessarily equate to one another, with some interventions being meritorious for both, while others only work for either *domain*. For example, chemo- and radiotherapy help to treat cancer but generally are not considered to be helpful for improving overall *health* status [18,19]. In contrast, good nutrition and physical activity do improve overall health and may aid in the recovery process from cancer but are generally not sufficient to stop established cancer from advancing on its own. There is a clearly overlooked opportunity for mental health care to mirror this parallel and to systematically adopt solutions with a broader focus than simply targeting or aiming to prevent and treat symptoms of mental illness. In other words, instead of an abundant reliance on reactive solutions, there may be a place for proactive solutions that *promote mental health and well-being* more broadly [16].

### Promotion of Mental Health vs Treatment of Mental Illness

Within mental health intervention research, there have been different streams seeking to promote mental health and well-being (eg, focusing on positive functioning and feeling well, high life satisfaction, more positive than negative emotions, a sense of purpose, and self-acceptance) [20]. These streams include proponents of dual-factor models [21], research on personal recovery [22], well-being therapy [23], positive (clinical) psychology [24,25], and positive psychiatry [26]. For decades, researchers working within these streams have built a considerable evidence base for psychological interventions for improving mental well-being [27-29]. For example, a recent systematic review of 419 studies (N=53,288) on psychological interventions to build mental well-being found that a wide range of interventions are beneficial in improving mental well-being but that the specific impact of intervention types depends on moderators, including the presence and severity of clinical symptoms [30]. For instance, interventions based on cognitive behavior therapy (CBT), which is well established in improving symptoms of mental illness [31,32], had a lesser impact on improving outcomes of mental well-being in nonclinical populations compared to clinical populations. On the contrary, interventions stemming from paradigms not specifically focused on treating symptoms of illness (eg, mindfulness and positive psychology interventions) were effective in improving mental well-being in nonclinical populations. Ultimately, the aforementioned review points to a sizeable evidence base that indicates the merit specific psychological interventions can have within stepped care models, particularly for those experiencing nonclinical symptoms of a mental illness. Specifically, such interventions promote mental well-being, which in turn can prevent the occurrence of mental illness as well as address symptoms of distress in early stages of common mental disorders (eg, depression and anxiety) [21,33,34].

As such, our team has developed an intervention that explicitly targets both mental health and well-being, the *Be Well Plan* [35]. The intervention introduces participants to a wide range of evidence-based psychological activities (eg, mindfulness, problem solving, self-compassion, thought challenging), which are derived from effective interventions identified from the aforementioned meta-analysis [30]. The majority of selected activities, including mindfulness-based activities, have also been found to be effective in improving symptoms of distress and common mental disorders. In contrast to more structured and manualized interventions, which often provide a set number of activities as part of a fixed program, participants in the *Be Well Plan* can choose from an activity bank with 30 different activities. Throughout the program, participants are supported in experimenting with different activities while learning which of them work well for them as individuals with their specific needs and preferences. Over a period of 5 weeks, participants are introduced to different methods to select and experiment with activities that are relevant to their unique needs. Before the start of the program, participants complete the *Be Well Tracker*, a measurement tool that assesses participants’ well-being, resilience, and distress. Depending on individual responses, participants receive a tailored report indicating areas of strength and vulnerability. During the second session, participants use their measurement results to select activities for areas they would like to work on.

A key feature of the *Be Well Plan* is that the program does not rely on mental health–trained clinicians. Instead, the program is designed to be delivered using a train-the-trainer methodology. Facilitators undergo a structured training schedule before they guide participants through the program. The program content and processes have been developed to allow inclusion of facilitators from a variety of different backgrounds, including peers of participants. This supports a high level of tailoring for different target groups as well as for the sustainable implementation and scalability of the program.

Given the tailored nature of the *Be Well Plan* that considers participants’ characteristics, needs, and preferences, there is the potential that the intervention also improves outcomes beyond mental health and well-being, such as self-efficacy, a sense of control, and cognitive flexibility. Although preliminary evidence for the *Be Well Plan* program’s impact has been established [36], there is a clear need now to establish its efficacy using a more robust methodological design.

### Scalable, Group-Facilitated, Internet-Based Mental Health Solutions

Internet-based solutions are an avenue to deliver scalable and effective mental health interventions without draining clinical resources from existing models of care [37]. Notably, the effectiveness of internet-based interventions for mental health problems has been widely established [38-40]. Although less is known about the long-term effects (eg, follow-ups of 2 years or longer) of these interventions, a recent meta-analysis summarizing studies that have examined the long-term effects of internet-supported CBT showed diminished but large effects sizes over an average follow-up period of 3 years [41]. Internet-based or web-based solutions often utilize pre-recorded content, are self-guided, or involve smartphone apps [42]. The past 2 decades have pointed to the utility of these “self-directed” interventions in mental health care at all levels, demonstrating improvements in outcomes of mental illness, as well as outcomes of mental health and mental well-being [43,44].

Although self-guided modalities can be effective, high dropout rates are commonly reported, and research has demonstrated that these interventions often require highly self-motivated participants and do not appeal to everyone [45,46]. For example, a meta-analysis based on individual patient data of 10 randomized controlled trials (RCTs) of self-guided web-based interventions for depression suggested that almost 60% of participants dropped out before completing half of the treatment modules, while less than 20% completed all treatment modules [47]. One solution to overcome these drawbacks is the utilization of a hybrid approach that uses technology to facilitate “active” in-person or group-based care. The most well known of these approaches is telehealth, where psychological therapy is delivered using teleconferencing software [48]. Although telehealth has been used for a long time [49], it has been predominantly used within rural and remote clinical populations. Similar to the delivery of clinical care, teleconferencing software can be used to deliver interventions focusing on the promotion of mental health and well-being. Videoconferencing software, such as Skype (Microsoft Corp.), Zoom (Zoom Video Communications), or Microsoft Teams, has experienced a huge uptake in recent years, proliferating during the COVID-19 pandemic [50], where the majority of the global population was forced to shift to remote working as a result of health restrictions. As such, many group-based programs were successfully delivered via the internet, whereas prior to the pandemic, the delivery of group programs was often met with considerable skepticism.

Internet-based interventions have the advantage of being accessible independent of location, which is particularly relevant at the moment where access to face-to-face interventions is limited due to lockdowns or quarantining as a result of the current pandemic [51]. Internet-based interventions can also counter some existing system inequities, as they facilitate access beyond metropolitan areas in rural and remote areas (with good internet access). In countries such as Australia, where internet penetration is over 90% [52], using a program such as the *Be Well Plan* via teleconferencing software is particularly valuable, as the program can be delivered by trained facilitators and does not rely on clinicians, which are already limited, particularly outside of metropolitan areas [53,54].

In addition, the *Be Well Plan* can successfully reach vulnerable populations, who may be isolated or struggling but are not ready or able to access contemporary services due to various barriers, such as cost or time. One example of such a vulnerable population is university students, who have been found to experience significantly higher levels of psychological distress compared to their peers. There is a sizeable body of research [55] investigating the mental health of university students, demonstrating high rates of mental health problems [56-58]. University students are often going through a phase of transition, are financially vulnerable, or are removed from their support systems at home [59], which increases their risk of experiencing psychological distress. Among others, these factors can account for why students experience such difficulties and why they are considered a key priority group to be targeted using innovative mental health and well-being interventions [60].

### Study Aims

This study aims to advance the literature in 2 ways. First, we aim to test the efficacy of the *Be Well Plan* with a vulnerable population (university students) in improving primary outcomes of mental well-being, resilience, anxiety, and depression and secondary outcomes of self-efficacy, a sense of control, and cognitive flexibility. Second, we aim to examine participants’ engagement and satisfaction with the *Be Well Plan* facilitated in group format using teleconferencing software.

## Methods

### Trial Design

A 2-arm RCT was conducted comparing an active intervention (*Be Well Plan*) with a waitlist control condition.

### Ethics Approval

The trial was approved by the local Human Research Ethics Committee (#2163) and registered with the Australian New Zealand Clinical Trial Registry (ACTRN12621000180819).

### Recruitment and Procedure

University students were recruited between August 2020 and April 2021 through emails, lectures, social media posts, posters, and flyers at a medium-size public university (~24,500 enrolled students in 2020) in Adelaide, Australia. Recruitment messaging focused on inviting students to participate in a new program that aimed to build their mental health and well-being. All enrolled students across the university were eligible to participate; no other eligibility criteria applied. Participants were not paid, and the study was not part of the university’s credit system to perform research. English language proficiency was assumed, as all students had passed the university language requirement for English before their university enrolment. Similarly, given the requirements of tertiary study, computer and internet literacy was assumed.

Participants registered their interest via an online survey and indicated their preferred day and time for the 5 intervention sessions. They could then attend a general information session about the content and structure of the program, hosted online via the teleconferencing software Zoom, or watch a pre-recorded version. After providing their informed consent electronically to participate in the trial, individuals completed an online baseline assessment, including general demographic questions (ie, age, gender, ethnicity, student and employment status) and their overall health status (ie, diet, activity, sleep), as well as primary (ie, well-being, resilience, depression, anxiety) and secondary outcome measures (self-efficacy, perceived sense of control, cognitive flexibility); see a detailed description for outcome measures later. The baseline survey included 170 questions, and the median completion time was 25 minutes.

After completing the baseline assessment, participants were randomized into either the intervention or the waitlist control group. As it was expected that some participants in the intervention group would not be able to commence the program due to unavailability, we chose a 2:1 allocation ratio for the intervention group. Randomization was stratified by gender, performed by a researcher who was not involved in the delivery of the intervention using a random number generator [61]. Participants in the intervention group took part in the weekly 5-session, group-based program, which was delivered online via Zoom and was accessible for students regardless of their study location (ie, students who were not physically located in Adelaide). The group sizes ranged from 18 to 26 participants, and in total, 10 individual groups were facilitated from August 2020 to June 2021. Participants from the waitlist control group gained access to the program after the intervention group; facilitators were not aware whether they were delivering the program to the intervention or the waitlist control group.

Next, 6 weeks after the baseline assessment, participants in both groups were asked to complete another online assessment including the primary and secondary outcome measures. This survey included 175 questions, and the median completion time was 23 minutes.

This meant participants from the intervention group completed the postintervention assessment 1 week after the final session of the program. Participants from the intervention group were also asked questions about their satisfaction with the program. Participants from the waitlist control group were given access to the intervention following their second assessment. Up to 3 email reminders were sent to participants to complete the assessment.

### Study Conditions

The study involved 2 conditions: the intervention group, which underwent the 5-week *Be Well Plan* program facilitated via Zoom, and the waitlist control group, which gained access to the *Be Well Plan* after the 5-week intervention period.

#### Be Well Plan Intervention

Participants allocated to the intervention group received detailed information, including the Zoom link to the first session of the *Be Well Plan*, prior to the start of the program. They further received a separate email inviting them to complete a brief 10-to-15-minute survey to assess their levels of mental health and well-being via a platform called the *Be Well Tracker*. The *Be Well Tracker* uses validated mental health and well-being scales: well-being and life satisfaction were measured using the Mental Health Continuum-Short Form [62] and the Satisfaction with Life Scale [63], respectively; resilience was measured with the Brief Resilience Scale [64]; and psychological distress was assessed using the Depression Anxiety Stress Scales [65]. The *Be Well Tracker* includes 50 items, and the median completion time for participants was 8 minutes. After completing the *Be Well Tracker*, participants received a detailed report about their levels of well-being, resilience, and distress, which provided them with relevant information that would be used throughout the *Be Well Plan* intervention. Thus, outcomes from the *Be Well Tracker* were solely used within the intervention and not for any analyses examining the efficacy of the program.

The *Be Well Plan* intervention has been previously described in a detailed paper by van Agteren et al [35], outlining the individual components of the program and providing insights into program materials, including screenshots of the intervention. In summary, the *Be Well Plan* is a weekly, 5-session internet-based, group-facilitated intervention that aims to improve mental health and well-being. The program assists participants in developing their own well-being plan tailored to their individual circumstances and needs. Participants learn and experiment with a range of evidence-based activities and skills targeted at improving mental health and well-being. Each session provides evidence-based information, self-reflection activities, and sharing of experiences between participants. The *Be Well Plan* introduces participants to an activity bank consisting of 30 evidence-based activities, which are selected from a large meta-analysis. Participants use various decision-making tools and visual aids (eg, flowcharts to find relevant activities based on self-reflection exercises). They are further supported by technology to find activities for their own unique needs. For example, they use their own results from *Be Well Tracker* measurements to find activities matched to their needs. Thus, participants can tailor the program according to particular needs and circumstances [30]; for a more detailed description of the individualization of the intervention, see the paper by van Agteren et al [35].

Each session was conducted via Zoom by 2 trained facilitators to ensure that the program adhered to the intervention protocol [35] and was delivered in an engaging and safe way. In total, 5 facilitators (authors KA, JvA, MI, and TM, and GF, AH, and KS) with a variety of professional backgrounds, including well-being research, counselling, and clinical psychology, delivered the program. A detailed description of the weekly content of the 5-week program can be found in Table S1 in Multimedia Appendix 1.

#### Waitlist Control Group

Participants in the waitlist control group were asked to complete the pre- and postintervention assessments, after which they were provided with access to the 5-week group-facilitated *Be Well Plan* sessions.

### Demographic Questions

At baseline, participants were asked about their age in years, gender (ie, male, female, nonbinary), ethnicity (ie, Caucasian, Asian/Indian, others/prefer not to say), student status (ie, domestic, international), and employment status (ie, part-/full-time, no employment, unemployed/lost job due to COVID-19, other). Furthermore, general health levels were assessed with the following questions: “In general, how would you say your health is?” ranked from 1 (*poor*) to 5 (*excellent*); “What best describes your activity level?” ranked from 1 (*seldom active, sedentary activities*) to 3 (*vigorously active for at least 30 minutes, 3 times a week*); and “Please report the quality of your sleep over the past 24 hours,” ranked from 1 (*worst-possible sleep*) to 12 (*best-possible sleep*).

### Outcome Measures

Primary outcome measures assessed participants’ well-being and resilience as well as their levels of depressive and anxiety symptoms. Secondary outcomes included self-efficacy, a sense of control, and cognitive flexibility.

#### Well-Being

The Warwick Edinburgh Mental Well-Being Scale (WEMWBS) was used to assess mental well-being, including eudaimonic and hedonic aspects of well-being [66]. The 14-item scale asks participants to indicate how often, over the past 2 weeks, from 0 (*none of the time*) to 5 (*all of the time*) they have experienced different thoughts and feelings (eg, “I’ve been feeling useful.”). Total scores range from 0 to 70, with higher scores indicating greater levels of mental well-being. Tennant et al [66] found that the WEMWBS demonstrates good content and construct validity, adequate test-retest reliability, and good internal consistency (Cronbach *α*=.83). The internal consistency of the WEMWBS in this study was excellent (Cronbach *α*=.91).

#### Resilience

The 10-item Connor-Davidson Resilience Scale (CD-RISC-10) [67] was used to assess resilience. Participants respond on a 5-point Likert scale from 0 (*not true at all*) to 4 (*true nearly all the time*) on how well they cope with adversity (eg, “I am able to adapt when changes occur.”). Total scores range from 0 to 40, with higher scores indicating greater levels of resilience. The CD-RISC-10 has demonstrated good construct validity and internal consistency (Cronbach *α*=.85) [68]. The internal consistency of the CD-RISC-10 in this study was good (Cronbach *α*=.88).

#### Depression

The 9-item Patient Health Questionnaire (PHQ-9) [69] was used to assess symptoms of depression. Participants respond on a 4-point Likert scale from 0 (*not at all*) to 3 (*nearly every day*) on how often they have experienced depressive symptoms (eg, “feeling tired or having little energy” or “feeling down, depressed, or hopeless”) over the past 2 weeks. Total scores range from 0 to 27, with higher scores indicating greater levels of depressive symptoms. The PHQ-9 has demonstrated good construct validity and internal consistency (Cronbach *α*=.86-.89) [69]. The internal consistency of the PHQ-9 in this sample was good (Cronbach *α*=.85). The following cut-offs were used in this study: 0-9=no-to-mild depressive symptoms and ≥10=moderate-to-severe depressive symptoms [69].

#### Anxiety

The 7-item General Anxiety Disorder (GAD-7) [70] was used to assess symptoms of anxiety. Participants respond on a 4-point Likert scale from 0 (*not at all*) to 3 (*nearly every day*) on how often they have experienced symptoms of anxiety (eg, “feeling nervous, anxious, or on edge” or “trouble relaxing”) over the past 2 weeks. Total scores range from 0 to 21, with higher scores indicating greater levels of anxiety. The GAD-7 has demonstrated good construct validity and internal consistency (Cronbach *α*=.91) [70]. The internal consistency of the GAD-7 in this sample was good (Cronbach *α*=.85). The following cut-offs were used in this study: 0-9=minimal-to-mild anxiety and ≥10=moderate-to-severe anxiety [70].

#### Self-efficacy

The 8-item New General Self-Efficacy Scale (NGSES) [71] was used to assess levels of general self-efficacy. Participants respond on a 5-point Likert scale from 1 (*strongly disagree*) to 5 (*strongly agree*) on how often they believe they can achieve their goals, despite difficulties (eg, “When facing difficult tasks, I am certain that I will accomplish them.”). Total scores range from 1 to 5, with higher scores indicating greater levels of self-efficacy. The NGSES has demonstrated good predictive validity and internal consistency (Cronbach *α*=.85-.91) [71]. The internal consistency of the NGSES in this sample was good (Cronbach *α*=.87).

#### Sense of Control

The 12-item Sense of Control Scale (SCS) [72] was used to assess participants’ perceived sense of control over their lives. Participants respond on a 7-point Likert scale from 1 (*strongly agree*) to 7 (*strongly disagree*) on how much they feel they can control (eg, “Whether or not I am able to get what I want is in my own hands.”) or not control (eg, “Other people determine most of what I can and cannot do.”) their personal lives. Total scores range from 1 to 7, with higher scores indicating greater control. The internal consistency of the SCS in this sample was good (Cronbach *α*=.84).

#### Cognitive Flexibility

The 12-item Cognitive Flexibility Scale (CFS) [73] was used to assess participants’ mental and cognitive flexibility. Participants respond on 6-point Likert scale from 1 (*strongly disagree*) to 6 (*strongly agree*) on how much they are aware of alternatives available to them (“I have many possible ways of behaving in any given situation.”) and their willingness and ability to be flexible and adapt to situations (“I am willing to work at creative solutions to problems.”). Total scores range from 12 to 72, with higher scores indicating greater cognitive flexibility. The CFS has demonstrated good construct validity and internal consistency (Cronbach *α*=.76-.77). The internal consistency of the CFS in this sample was acceptable (Cronbach *α*=.77).

#### Engagement

Engagement with the program was assessed by recording whether participants attended the individual *Be Well Plan* sessions. Perceived session engagement was assessed after each session with a single item (“I felt engaged in the session.”). Participants responded on a 5-point Likert scale from 1 (*strongly disagree*) to 5 (*strongly agree*).

#### Satisfaction

Participants’ satisfaction with the program was assessed with a single item (“Overall, how satisfied were you with the program?”) during the postintervention assessment. Participants responded on a 5-point Likert scale from 1 (*very dissatisfied*) to 5 (*very satisfied*). Satisfaction with the individual *Be Well Plan* sessions was assessed after each session with a single item (“Overall, how satisfied were you with the session?”). Again, participants responded on a 5-point Likert scale from 1 (*very dissatisfied*) to 5 (*very satisfied*).

### Statistical Analyses

Data were collected online using Qualtrics [74]. Data analyses were performed using IBM SPSS Statistics v27 [75]. For all analyses, a significance level of Cronbach *α*=.05 was applied. Cohen *d* was calculated for both between- and within-subject effect sizes using the following formulae:

d = (M_1_ – M_2_)/SD_pooled_ (between subjects),

SD_pooled_ = √[(S_1_^2^ + S_2_^2^)/2],

d = (M_diff_)/SD_diff_ (within subjects),

SD_diff_ = √[(S_1_^2^ + S_2_^2^) – 2 × r_12_ × S_1_ × S_2_,

where M_1_ and M_2_ are the means of the intervention and the waitlist control group, respectively; SD is the standard deviation; and r_12_ is the correlation between the intervention and the waitlist control group.

Pre- and postintervention differences were analyzed with mixed ANOVAs in both modified intention-to-treat (mITT; n=75, 59.5%) and completer (n=49, 38.9%) samples. All participants from the intervention group who participated in at least 1 *Be Well Plan* session were included in the mITT [76]. The Little missing completely at random (MCAR) test was performed to test whether data were missing completely at random (*χ*^2^_11_=14.23, *P*=.22), suggesting missing data in the 7 outcomes measures were missing completely at random. Thus, for the mITT analyses, we imputed missing data on the outcome measures for participants who did not complete the postintervention assessment (n=61, 37.2% of the analyzed sample, N=164) using a Markov chain Monte Carlo method and information from the following variables: gender, age, ethnicity, working and student status, and pre- and postintervention scores in well-being, resilience, depression, anxiety, self-efficacy, a sense of control, and cognitive flexibility. In total, we simulated 10 new data sets using a maximum of 100 iterations from which mean scores for postintervention outcomes were computed and used for the mITT analyses. As there were small but significant age differences between the intervention and waitlist control groups, we additionally performed separate mixed ANOVAs for all outcome measures while controlling for age in years. As age was a nonsignificant contributor in any of the analyses, the following results are presented without age as a covariate.

Reliable change analysis was conducted by calculating a reliable change index (RCI) using the method suggested by Jacobson and Truax [77]. Separate RCIs for the mITT and completer samples were calculated by subtracting participants’ postintervention scores from their baseline and subsequently dividing this difference score by the SE of the difference for the measurements used. The SE of the difference was estimated by

SE_diff_ = SD_x_ × √(1 – r_xx_),

where SD_x_ refers to the SD of the difference scores and r_xx_ refers to the internal consistency of the measure (ie, Cronbach *α*). Any change larger than 1.96 was considered reliable.

## Results

### Participants

**Figure 1:**
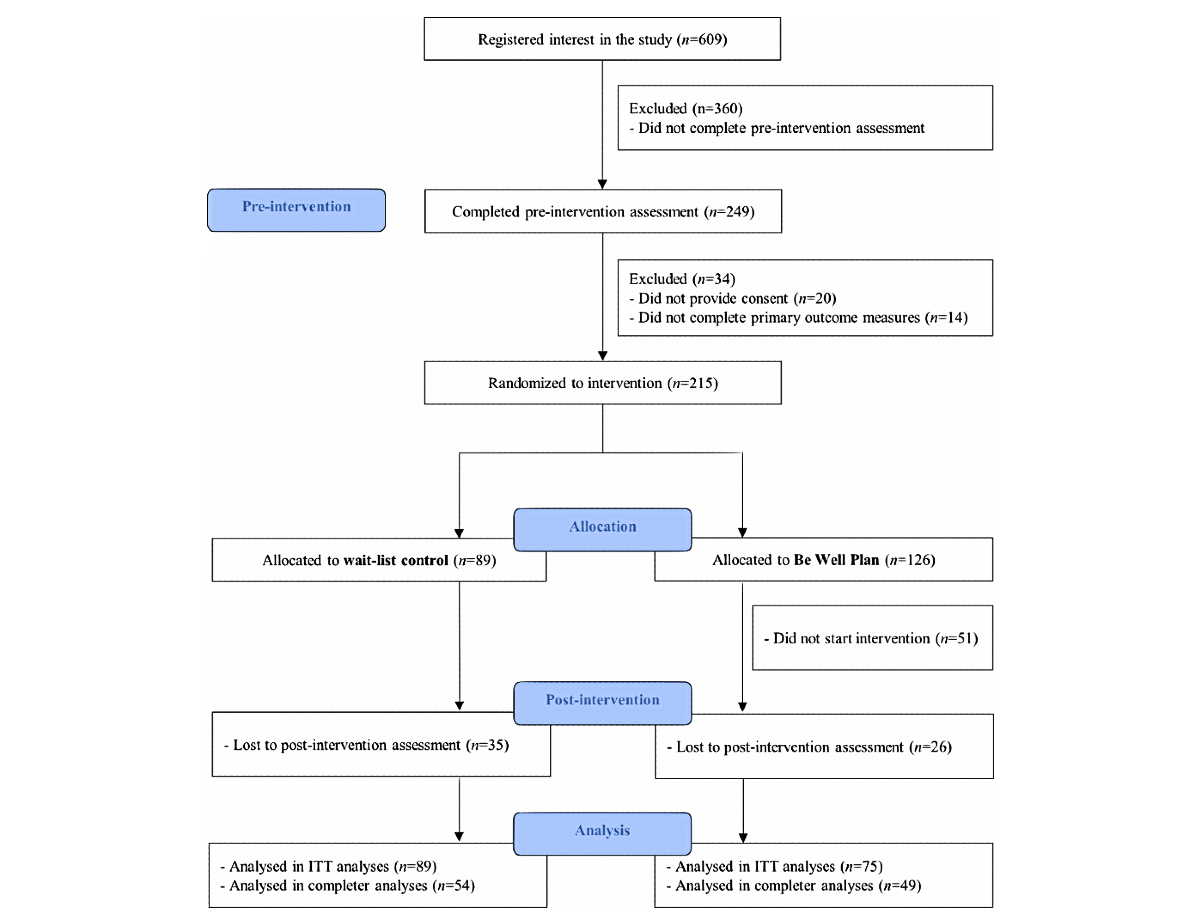
Consolidated Standards of Reporting Trials (CONSORT) flow diagram of study. mITT: modified intention to treat.

The participant flow is shown in Figure 1. Based on an a priori power analysis [78], we estimated a sample size of 202 participants: statistical power=.80, Cronbach *α*=.05, Cohen *d*=0.5, and 40% attrition with a 2:1 allocation ratio for the intervention group. A total of 215 participants were randomized to the intervention (n=126, 58.6%) or the waitlist control (n=89, 41.4%) group. Of the 126 participants who were allocated to the *Be Well Plan* condition, 51 (40.5%) participants did not commence the program. Participants who did not commence the program reported other time commitments or unavailability for the scheduled session time (n=44, 86.3%). There were no significant differences between participants from the intervention group who attended at least 1 *Be Well Plan* session (n=75, 59.5%) and those who did not commence the program on any of the baseline outcome measures or demographic variables except for age; participants who did not commence with the program were, on average, 5.88 years younger (*F*_1,125_=9.81, *P*=.002). For the waitlist control group, there were no significant differences between those participants who completed (n=54, 61%) versus those who did not complete the postassessment (n=35, 39%); all *P*>.24.

Next, we report demographic information about the 75 participants from the intervention group who attended at least 1 *Be Well Plan* session and the 89 participants who were allocated to the waitlist control group; see Tables 1 and 2. On average, participants were 30.65 years old (SD 10.10), with the majority being female (133/164, 81.1%), Caucasian (94/164, 57.9%; 48/164, 29.3%, were Asian/Indian), and domestic (122/164, 74.4%) students. The majority of participants were employed (part- or full-time, 87/164, 53.0%), while others did not work (42/164, 25.6%) or were unemployed/had lost their job due to COVID-19 (25/164, 15.2%). On average, participants rated their overall health (mean 2.81, SD 1.03) and their diet as *fair*/*good* (mean 2.78, SD 1.06), their activity level as moderate (mean 1.76, SD 0.72), and their sleep quality as *fair*/*good* (mean 7.35, SD 2.78). Almost half of the sample (n=81, 49.4%) reported moderate-to-severe levels of depression, while over one-third (n=59, 36%) reported moderate-to-severe levels of anxiety, suggesting the vulnerability of this student cohort.

There were no significant differences between intervention and waitlist control groups on gender and ethnicity, nor were there differences on student or employment status or health status. However, there was a small but significant difference in age between the 2 groups; participants allocated to the *Be Well Plan* intervention were, on average, 3.11 years older compared to participants in the waitlist control group.

**Table 1:** Participants’ demographics and preintervention characteristics (*F* test).

Variable	Be Well Plan (N=75)		Waitlist control (N=89)		Significance statistics	
	n (%)	Mean (SD)	n (%)	Mean (SD)	*F_df_*	*P* value
Age	75 (100)	32.33 (10.59)	89 (100)	29.22 (9.50)	*F*_1,162_=3.93	.05
Overall health	74 (99)	2.77 (1.05)	87 (98)	2.85 (1.01)	*F*_1,159_=0.24	.62
Diet	74 (99)	2.70 (0.99)	87 (98)	2.85 (1.12)	*F*_1,159_=0.78	.38
Activity level	74 (99)	1.78 (0.73)	87 (98)	1.75 (0.72)	*F*_1,159_=0.10	.75
Sleep	74 (99)	7.19 (2.64)	87 (98)	7.49 (2.91)	*F*_1,159_=0.48	.49

**Table 2:** Participants’ demographics and preintervention characteristics (chi-square test).

Variable		Be Well Plan (N=75)	Waitlist control (N=89)
		n (%)	n (%)
**Gender^a^ (*χ*^2^_1_=0.10, *P*=.92)**			
	Male	13 (17.3)	16 (18.0)
	Female	61 (81.3)	72 (80.9)
	Nonbinary	1 (1.3)	1 (1.1)
**Ethnicity (*χ*^2^_2_=3.11, *P*=.21)**			
	Caucasian	49 (65.3)	46 (51.7)
	Asian/Indian	18 (24.0)	30 (33.7)
	Others/prefer not to say	8 (10.7)	13 (14.6)
**Student status (*χ*^2^_1_=1.72, *P*=.19)**			
	Domestic	59 (78.7)	63 (70.8)
	International	16 (21.3)	26 (29.2)
**Employment status (*χ*^2^_3_=1.34, *P*=.72)**			
	Part-/full-time	40 (53.3)	47 (52.8)
	No	17 (22.7)	25 (28.1)
	Unemployed/lost job due to COVID-19	12 (16.0)	13 (14.6)
	Other	6 (8.0)	4 (4.5)

^a^Comparison conducted only for male vs female.

### Participant Engagement and Satisfaction With the Program

Participants in the intervention group (ie, n=75, 59.5%, who attended at least 1 session) attended, on average, 3.41 sessions (SD 1.56, median 4); 55 (73.3%) attended at least 4 sessions, and 25 (33.3%) attended all 5 sessions. Those included in the completer analyses (n=49, 38.9%, who attended at least 1 session and completed pre- and postintervention assessments) attended, on average, 4.20 sessions (SD 1.00, median 4); 41 (83.7%) of the participants attended at least 4 sessions, and 23 (46.9%) attended all 5 sessions.

Of the 49 intervention group participants who completed the postintervention assessment, 47 (95.9%) were either very satisfied (n=31, 66%) or satisfied (n=16, 34%). Session feedback was available from 28 (37.3%, session 5) to 57 (76%, session 1; overall median response rate=39) of 75 participants across the 5 *Be Well Plan* sessions. Overall, 68-75 (91.2%-100%) of participants felt engaged during the sessions, while 63-75 (84.2%-100%) participants were either satisfied or very satisfied with the quality of the sessions.

### Primary Outcomes

A detailed outline of the scores for the *Be Well Plan* and waitlist control groups for the primary and secondary outcomes, including effect sizes, can be found in Tables 3 and 4. Reliable change analysis showed that the majority (58/75, 77.3%) of the mITT participants demonstrated a significant reliable improvement in at least 1 of the primary outcomes. When looking at data for the completer sample, we found that a vast majority (40/49, 81.6%) of participants showed a reliable change in at least 1 outcome.

**Table 3:** Primary outcomes (well-being, resilience, depression, and anxiety): mITT^a^ analysis.

Time		Be Well Plan (N=75)		Waitlist control (N=89)		Effects size, Cohen *d* (95% CI)			
		Mean (SD)	SE	Mean (SD)	SE	Within groups		Between groups	
**Well-Being**							0.65 (0.40-0.90)		0.49 (0.17-0.80)
	Preintervention	40.44 (8.61)	0.99	43.43 (9.89)	1.05	N/A^b^		N/A	
	Postintervention	46.12 (8.41)	0.97	44.74 (6.99)	0.74	N/A		N/A	
**Resilience**							0.46 (0.22-0.69)		0.44 (0.13-0.75)
	Preintervention	22.93 (5.72)	0.66	24.27 (6.30)	0.67	N/A		N/A	
	Postintervention	25.68 (5.52)	0.64	24.52 (5.54)	0.59	N/A		N/A	
**Depression**							0.66 (0.41-0.90)		0.32 (0.01-0.63)
	Preintervention	10.95 (5.81)	0.67	9.67 (5.30)	0.56	N/A		N/A	
	Postintervention	7.70 (4.36)	0.50	8.02 (3.60)	0.38	N/A		N/A	
**Anxiety**							0.58 (0.33-0.82)		0.37 (0.06-0.68)
	Preintervention	9.27 (5.06)	0.58	8.15 (4.57)	0.48	N/A		N/A	
	Postintervention	6.46 (4.08)	0.47	7.09 (4.11)	0.44	N/A		N/A	

^a^mITT: modified intention to treat.

^b^N/A: not applicable.

**Table 4:** Primary outcomes (well-being, resilience, depression, and anxiety): completer analysis.

Time		Be Well Plan (N=49)		Waitlist control (N=54)		Effects size, Cohen *d* (95% CI)			
		Mean (SD)	SE	Mean (SD)	SE	Within groups		Between groups	
**Well-Being**							0.77 (0.44-1.08)		0.66 (0.26-1.06)
	Preintervention	40.35 (8.55)	1.22	43.74 (10.24)	1.39	N/A^a^		N/A	
	Postintervention	46.73 (10.24)	1.46	44.37 (8.94)	1.22	N/A		N/A	
**Resilience**							0.58 (0.27-0.88)		0.76 (0.36-1.16)
	Preintervention	22.63 (5.20)	0.74	24.83 (6.37)	0.87	N/A		N/A	
	Postintervention	25.92 (6.81)	0.97	24.20 (7.08)	0.96	N/A		N/A	
**Depression**							0.79 (0.46-1.11)		0.39 (–0.01 to 0.78)
	Preintervention	11.08 (5.86)	0.84	9.74 (5.31)	0.72	N/A		N/A	
	Postintervention	7.61 (5.37)	0.77	8.09 (4.61)	0.63	N/A		N/A	
**Anxiety**							0.56 (0.25-0.86)		0.28 (–0.11 to 0.67)
	Preintervention	8.69 (4.83)	0.69	8.33 (4.71)	0.64	N/A		N/A	
	Postintervention	6.20 (5.02)	0.72	7.15 (5.26)	0.72	N/A		N/A	

^a^N/A: not applicable.

#### Well-Being

The mITT analysis for well-being showed a significant time × group interaction effect (*F*_1,162_=9.65, *P*=.002) and a significant main effect of time (*F*_1,162_=24.77, *P*<.001); however, there was no significant main effect of group (*F*_1,162_=0.50, *P*=.48). The results suggest that both groups improved over time, but the *Be Well Plan* group showed significant greater improvement compared to the waitlist control group. These results were replicated with the completer analysis: time × group interaction effect (*F*_1,101_=11.19, *P*=.001); main effect of time (*F*_1,101_=16.62, *P*<.001); and main effect of group (*F*_1,101_=0.10, *P*=.76).

#### Resilience

The mITT analysis for resilience showed a significant time × group interaction effect (*F*_1,162_=7.85, *P*=.01) and a significant main effect of time (*F*_1,162_=11.35, *P*<.001); however, there was no significant main effect of group (*F*_1,162_=0.01, *P*=.91). The results, similar to the pattern found for well-being, suggest that the *Be Well Plan* group showed significantly greater improvement in resilience compared to the waitlist control group. Results were again replicated in the completer sample: time × group interaction effect (*F*_1,101_=14.91, *P*<.001); main effect of time (*F*_1,101_=6.86, *P*=.01); and main effect of group (*F*_1,101_=0.04, *P*=.84).

#### Depression

The mITT analysis for depression showed again a significant time × group interaction effect (*F*_1,162_=4.14, *P*=.04) and a significant main effect of time (*F*_1,162_=39.64, *P*<.001); however, there was no significant main effect of group (*F*_1,162_=0.55, *P*=.46). Therefore, significantly greater improvements in depression were noted for the *Be Well Plan* group compared to the waitlist control group. Results were not replicated in the completer sample, as the time × group interaction effect (*F*_1,101_=3.88, *P*=.05) did not meet our significance threshold; similar to the mITT analysis, there was a significant main effect of time (*F*_1,101_=30.60, *P*<.001) and no significant main effect of group (*F*_1,101_=0.21, *P*=.65).

#### Anxiety

Similarly to depression, the mITT analysis for anxiety showed a significant time × group interaction effect (*F*_1,162_=5.64, *P*=.02) and a significant main effect of time (*F*_1,162_=27.41, *P*<.001); however, there was no main effect of group (*F*_1,162_=0.17, *P*=.68). Results thus indicate that the *Be Well Plan* group improved more in anxiety symptoms compared to the waitlist control group. Results from the completer analysis differed as the time × group interaction effect (*F*_1,101_=2.01, *P*=.16) was not statistically significant; similar to the mITT analysis, there was a main effect of time (*F*_1,101_=15.97, *P*<.001) and no significant main effect of group (*F*_1,101_=0.11, *P*=.74).

### Differential Change in Primary Outcomes

Of the total mITT participants who demonstrated a reliable change in depression or mental well-being, most (27/48, 56.3%) only showed a change in well-being, with 8 (16.7%) only demonstrating a change in depression and 13 (27.1%) demonstrating a change in both outcomes. Of the 48 participants who demonstrated a change in well-being and anxiety, most (25/48, 52.1%) showed a change in both outcomes, with 8 (16.7%) only demonstrating a change in anxiety and 15 (31.3%) only showing a change in mental well-being.

Results were similar for the completer analysis. For participants who had a change in mental well-being and depression, the majority (17/33, 51.5%) improved in both outcomes, with 7 (21.2%) only improving in depression and 9 (27.3%) only improving in mental well-being. Of the 31 participants who demonstrated a reliable change in mental well-being and anxiety, the majority (16/31, 51.6%) showed a reliable change in both outcomes, with 5 (16.1%) only showing a change in anxiety and 10 (32.3%) only showing a change in mental well-being.

Only 5 participants (mITT: 5/75, 6.7%; completer: 5/49, 10.2%) reported a reliable deterioration in well-being, depression, or anxiety: 1 participant showed a reliable decrease in well-being (mITT: 1/75, 1.3%), whereas 2 participants showed a reliable increase in depression or anxiety symptoms (mITT: 4/75, 5.3%). Importantly, no participant showed reliable deterioration in more than 1 of the mentioned outcomes. Furthermore, no participant reported any harmful effects from participation in the program.

### Secondary Outcomes

#### Self-efficacy

The mITT analysis for self-efficacy showed a significant time × group interaction effect (*F*_1,162_=4.00, *P*=.047) and a significant main effect of time (*F*_1,162_=10.75, *P*=.001); however, there was no main effect of group (*F*_1,162_=0.42, *P*=.52); see Tables 5 and 6. Results therefore suggest that participants in the *Be Well Plan* group increased more in self-efficacy compared to the waitlist control group. Results differed in the completer analysis: although the time × group interaction effect was still significant (*F*_1,99_=6.75, *P*=.01), the main effect of time (*F*_1,99_=3.83, *P*=.05) and group (*F*_1,99_=0.72, *P*=.40) was not.

**Table 5:** Secondary outcomes (self-efficacy, sense of control, and cognitive flexibility): mITT^a^ analysis.

Time		Be Well Plan (N=75)		Waitlist control (N=89)		Effects size, Cohen *d* (95% CI)			
		Mean (SD)	SE	Mean (SD)	SE	Within groups		Between groups	
**Self-efficacy**							0.41 (0.17-0.65)		0.31 (0.01-0.62)
	Preintervention	3.55 (0.60)	0.07	3.69 (0.62)	0.07	N/A^b^		N/A	
	Postintervention	3.80 (0.51)	0.07	3.75 (0.55)	0.08	N/A		N/A	
**Sense of control**							0.38 (0.15-0.62)		0.36 (0.05-0.67)
	Preintervention	4.65 (0.90)	0.10	4.89 (0.94)	0.10	N/A		N/A	
	Postintervention	5.03 (0.88)	0.12	4.96 (0.81)	0.10	N/A		N/A	
**Cognitive flexibility**							0.47 (0.23-0.71)		0.36 (0.05-0.67)
	Preintervention	51.54 (6.16)	0.71	53.51 (7.20)	0.76	N/A		N/A	
	Postintervention	54.69 (5.42)	0.65	54.28 (5.94)	0.65	N/A		N/A	

^a^mITT: modified intention to treat.

^b^N/A: not applicable.

**Table 6:** Secondary outcomes (self-efficacy, sense of control, and cognitive flexibility): completer analysis.

Time		Be Well Plan (N=49)		Waitlist control (N=54)		Effects size, Cohen *d* (95% CI)			
		Mean (SD)	SE	Mean (SD)	SE	Within groups		Between groups	
**Self-efficacy**							0.48 (0.18-0.76)		0.50 (0.11-0.90)
	Preintervention	3.54 (0.55)	0.08	3.79 (0.63)	0.09	N/A^a^		N/A	
	Postintervention	3.81 (0.61)	0.09	3.75 (0.65)	0.09	N/A		N/A	
**Sense of control**							0.36 (0.07-0.65)		0.42 (0.03-0.82)
	Preintervention	4.66 (0.81)	0.12	4.98 (0.98)	0.13	N/A		N/A	
	Postintervention	5.02 (1.07)	0.15	4.98 (1.03)	0.14	N/A		N/A	
**Cognitive flexibility**							0.47 (0.17-0.76)		0.65 (0.25-1.05)
	Preintervention	51.69 (5.91)	0.84	54.89 (7.22)	0.97	N/A		N/A	
	Postintervention	54.79 (6.78)	0.98	53.98 (7.69)	1.06	N/A		N/A	

^a^N/A: not applicable.

#### Sense of Control

The mITT analysis for sense of control showed a significant time × group interaction effect (*F*_1,162_=5.15, *P*=.03) and a significant main effect of time (*F*_1,162_=10.76, *P*=.001); however, there was no main effect of group (*F*_1,162_=0.59, *P*=.45). These results indicate that participants in the *Be Well Plan* group increased more in their sense of control compared to the waitlist control group. Results were replicated in the completer analysis: time × group interaction effect (*F*_1,99_=4.53, *P*=.04), main effect of time (*F*_1,99_=4.22, *P*=.04), and main effect of group (*F*_1,99_=0.61, *P*=.44).

#### Cognitive Flexibility

The mITT analysis for cognitive flexibility showed a significant time × group interaction effect (*F*_1,162_=5.39, *P*=.02) and a significant main effect of time (*F*_1,162_=14.65, *P*<.001); however, there was no main effect of group (*F*_1,162_=0.87, *P*=.35). These findings indicate that participants in the *Be Well Plan* group increased more in cognitive flexibility compared to the waitlist control group. Results differed in the completer analysis: although the time × group interaction effect was still significant (*F*_1,99_=10.75, *P*=.001), the main effect of time (*F*_1,99_=3.23, *P*=.08) and group (*F*_1,99_=0.92, *P*=.40) was not.

## Discussion

### Principal Findings

This study examined the efficacy of a group-facilitated, internet-based program to promote mental health and well-being in a vulnerable population of university students. Compared to waitlist controls, participants in the intervention group significantly improved in all primary outcomes, including mental well-being, resilience, depression, and anxiety, as well as secondary outcomes, including self-efficacy, a sense of control, and cognitive flexibility. Furthermore, participants’ engagement and satisfaction with the *Be Well Plan* were examined, showing that students were highly engaged and satisfied with the program.

### Improvements in Well-Being and Resilience

The study clearly demonstrated the anticipated significant improvements in mental well-being and resilience, confirming preliminary positive effects identified in a previous uncontrolled intervention study [36]. We found medium effect sizes for mental well-being, which is above the average typically reported in the literature [29,79]. For example, previous meta-analyses of psychological interventions in the general population reported small effect sizes for well-being interventions of similar intensity: programs longer than 4 weeks tend to produce small, positive effects according to van Agteren et al [30] (Hedges *g*=0.32), Sin and Lyubomirsky [80] (*r*=.36 for 5-7-week interventions). Importantly, effect sizes tend to be much lower for internet-based interventions (Hedges *g*=0.22) [eg, 30], attesting to the positive impact of the *Be Well Plan* program. For resilience, we found small-to-medium effect sizes, which is in line with what is typically reported in the research literature. For example, a meta-analysis of 11 RCTs examining resilience interventions by Joyce et al [81] reported a standardized mean difference of 0.44 between resilience interventions and waitlist control groups.

There are various potential sources for the observed positive effects. First, the program was rigorously designed based on a best-practice intervention development methodology [35]. The intervention-mapping approach [82] and comparable development methodologies, such as the behavior change wheel [83], are frequently used in health promotion research but not readily in mental health or psychology research. Using the intervention-mapping process meant that the program was (1) designed based on a comprehensive needs analysis, (2) grounded in a well-defined theory of behavior change, (3) co-designed using knowledge and experience from a range of different stakeholders (ie, psychologists, counsellors, mental health researchers, end users), and (4) composed of evidence-based behavior change techniques. The included activities were based on our team’s research into effective well-being interventions [30], resulting in activities from a wide variety of therapeutic approaches, including CBT, acceptance- and commitment-based therapy (ACT), mindfulness, and positive psychology. This “theory-agnostic” approach provides further explanation for the observed positive effects across the outcomes of well-being, resilience, depression, and anxiety, with these approaches having solid evidence for being able to change these outcomes [28,31,33,34,81].

Second, the intervention’s focus on tailoring and individualizing a well-being strategy to a participant’s unique context, needs, and preferences likely aided in achieving positive effects across all outcomes. In contrast to “generic” interventions, which are typically similar for all participants (ie, everyone receives the same content), the *Be Well Plan* was designed to allow participants to experiment with different techniques they wanted to include in their own well-being program (ie, *their Be Well Plan*) based on their perceived characteristics and needs. Previous research has argued for the importance of person-intervention fit through tailoring and individualization of programs or their components as a potential strategy to improve the efficacy of and engagement with psychological interventions, not just for clinical mental health programs, but also for well-being and mental health promotion programs [84-86]. Personalization plays a crucial role in face-to-face therapy but is similarly touted as an important advantage for internet-based interventions (eg, to increase personal relevance and engage users) [87-89]. Although the importance of tailoring interventions to individual characteristics, needs, and preferences has been highlighted in previous research [90], tailored interventions such as the *Be Well Plan*, which center around individual agency, are rare. The program allows individuals to choose their own activities and tailor these to their specific needs and preferences, fostering individual agency and autonomy, which is an important factor in improving health behaviors, including mental health and well-being, and an important component of contemporary well-being theories [91,92]. It is important to note that although this study’s purpose was not to investigate the superiority or noninferiority of our tailored approach over generic programs, research should look further into the impact of higher degrees of personalization on both efficacy and engagement in group-based programs.

Third, the facilitated group-based format of the *Be Well Plan* offered several advantages over a self-guided, individual approach, which likely improved both outcomes and engagement with the program. For example, sharing personal experiences in a safe and supportive environment may have led to vicarious learning and a feeling of being supported by others [93], while the trained facilitators guiding participants through the program and supporting them possibly increased engagement [94,95]. Importantly, the aspect of social connectedness has also been highlighted as a facilitator for user engagement in a recent systematic review [89]. Furthermore, allowing participants in internet-based group interventions to experiment with different evidence-based techniques in an effective manner has become much more within reach with the rise of technology [96,97]. For instance, technology can help guide activity recommendations based on an individual’s response to scientific questionnaires for mental health and well-being, aiding in personalization, as is the case for the *Be Well Plan*.

### Improvements in Depression and Anxiety

The positive effects on outcomes of depression and anxiety are encouraging, particularly as they build on similar outcomes found in a previous uncontrolled study of the intervention [36]. Psychological distress is an independent outcome to clinical symptoms [98]; therefore, finding improvements in both markers across the 2 studies points to the potential utility of the program for clinical settings. Although within-subject effect sizes were medium, between-subject effects were small. However, it is important to note that the recruited cohort was not a clinical sample, implying that effects could potentially be greater in individuals with clinical depression or anxiety. Having said that, university students are known to be an at-risk population reporting poor mental health, including depression and anxiety [56-58], which was shown by the high proportion of participants reporting moderate-to-severe baseline levels of depression and anxiety.

Our findings also need to be interpreted in the context of the worldwide COVID-19 pandemic. Recruitment and program participation took place between August 2020 and June 2021. Although the effects of COVID-19 in South Australia where the sample was recruited from were modest compared to other jurisdictions in Australia or worldwide, restrictions due to COVID-19 were still in place throughout the study period. For example, in March 2020, the South Australian government declared a public health emergency, which included measures such as closures of state borders and physical distancing requirements (eg, a 3-day lockdown in November 2020). Unsurprisingly, previous studies have found detrimental effects of COVID-19 on mental health in large, representative Australian cohorts [99,100], individuals who have been impacted by the adverse border closure effects of COVID-19 [101], and university students [102,103]. Thus, it is noteworthy that although the *Be Well Plan* did not directly target symptoms of depression or anxiety, almost half of the sample (46.7% for depression, 44.0% for anxiety) showed a reliable change in the respective outcomes.

### Improvements in Self-efficacy, Sense of Control, and Cognitive Flexibility

After participating in the *Be Well Plan*, improvements in self-efficacy, sense of control, and cognitive flexibility were observed. It might be that the tailored nature of the intervention, which encourages individuals to initially understand their own mental health and well-being and subsequently identify effective strategies to improve or maintain good levels of mental health, elicited the belief in individuals that they are able to change or take control of their life and can adapt to circumstances. This is important as self-efficacy, a sense of control, and cognitive flexibility have been identified as protective factors for good mental health [104-106].

### Participant Program Engagement and Satisfaction With the Program

Overall, engagement with the *Be Well Plan* was strong. Participants who began the program attended, on average, over two-thirds of the 5 sessions; of those who completed the postsession feedback, over 90% (n=68-75, 91.2%-100%) felt engaged during the sessions. The field of internet-based mental health interventions has been grappling with high attrition and dropout rates [107,108], particularly with fully self-guided programs and open-access trials. For this study, it is likely that the facilitated group-based setting of the *Be Well Plan* partly led to high participant engagement. In this regard, the *Be Well Plan* program was more akin to telehealth sessions—notably without using clinical resources—supporting participants much more than a fully self-guided internet-based intervention.

As previously discussed, a program feature is that participants experiment with different activities and techniques to build and personalize their own program. This personalization may have also led to high engagement and satisfaction with the program [84,85]. For example, over 47 (95.9%) participants were satisfied with the *Be Well Plan* program, while satisfaction rates with individual sessions ranged from n=63-75 (84.2%-100%).

A significant proportion of participants allocated to the intervention group (n=51, 40.5%) did not commence the *Be Well Plan*. The majority of those participants (n=44, 86.3%) reported other time commitments or unavailability for the scheduled session time. Although we found no significant differences between participants who commenced and did not commence the program in any of the baseline outcome measures or demographic variables—except for participants who did not commence the program, being younger—a potential for selection bias cannot be completely ruled out. Thus, participants who commenced the program were possibly more motivated from the outset.

### Strengths and Limitations

A major strength of this study was the pre-registered, rigorous RCT design. The RCT was conducted in a vulnerable population with high attendance rates and whose mental health benefitted from the intervention. This is 1 of the first studies to rigorously evaluate an online group-facilitated mental health intervention via teleconferencing that aims to improve both mental health and well-being without targeting specific symptoms or a specific group. The intervention is unique as it allows individuals to experiment with a variety of activities that can be tailored to their individuals needs and circumstances and encourages habit formation. A particular strength of the study was that symptoms of depression and anxiety were reduced, even though the *Be Well Plan* was not developed to specifically address these outcomes, nor were participants provided with any traditional psychoeducational information about these mental health problems.

Another strength was the web-based format of the intervention, which is particularly interesting in vast countries, such as Australia, where internet access is sufficient in regional and rural areas and mental health services are accepted and actively sought out [109]. Web-based interventions have previously been well accepted, allowing participants to interact while remaining in their own homes [110]. This modality has been investigated in the COVID-19 era, with interventions demonstrating efficacy, participant satisfaction, and engagement, while removing barriers and inconveniences related to attending in-person sessions [111,112].

Although there were several strengths of the study, some limitations need to be discussed. First, the study used a waitlist control group for comparison; although waitlist groups are cost-effective and ethical alternative control conditions, they might exaggerate effects sizes compared to other control conditions (eg, no intervention or active psychological placebo conditions) [113]. Future studies should test the *Be Well Plan* against an active, psychological placebo control group. Second, although the study population (ie, university students) was a vulnerable group, it does limit the generalizability of the findings to the general public. For instance, the general public typically reports better mental health, which in turn means that effect sizes in the general public may be considerably lower. Digital literacy may also be higher in a student population, potentially affecting accessibility and scalability for a broader range of specific or general community groups. Future work should be conducted to further test the *Be Well Plan* in different population cohorts. Third, the study was not sufficiently powered to find significant effects for depression and anxiety in the completer analysis, despite medium effect sizes. Although almost half of participants showed reliable improvements in depression and anxiety outcomes, future studies should include larger samples to allow to test for small-to-medium effects sies in psychological distress with clinical samples. Fourth, there was a high number of participants who registered their interest, completed the baseline measures, and were randomized to the intervention group but did not commence the first session. Although a limitation, this is common with internet-based programs; for example, a variety of studies in the area have noted similar engagement (uptake and adherence) challenges [114-116]. Fifth, our findings are short-term results only as our postassessment was taken at 1 week after the final session. The literature clearly indicates that the impact of mental health interventions diminishes over time, particularly in general well-being programs. Although investigating the long-term impact might look like an interesting question, the literature on diminishing returns is well established [30]. Rather, it is arguably more important to invest effort in designing and testing sustainable booster material [117]. Development work is currently underway to develop ongoing topical booster sessions that aim to both reinforce core program learnings and introduce new content and activities over time. Another limitation of this study was that we did not collect data on which activities individuals used during their participation in the program. Future studies should examine intervention processes (eg, which activities were used and how frequently) to better understand mechanisms of change. Furthermore, most participants were female. Although males who did participate in the program benefitted equally as female participants, some caution is required when generalizing results in males, due to a small sample size. Future studies need to attract and evaluate more males in programs such as the *Be Well Plan*.

Finally, as is the case with most psychological treatment studies, the outcome measures were all based on subjective reports. Future studies could feasibly undertake evaluations using behavioral or other objective measures. For instance, the use of technology can now facilitate evaluations using objective measures, such as activity levels, sleep patterns and other physiological outcomes, the use of health services, and prescribed psychopharmaceuticals. Knowing whether the *Be Well Plan* also advances improvements in such outcomes would add to its utility as a prevention and early intervention program.

### Conclusion

This is 1 of the first studies to rigorously evaluate a live-facilitated (eg, via teleconferencing software), online intervention, the *Be Well Plan*, that aims to improve both mental health and well-being without targeting specific symptoms or a particular target group. The intervention is unique as it allows individuals to experiment with a variety of activities that can be tailored to their individuals needs and circumstances and encourages habit formation. A particular strength of the study was that symptoms of depression and anxiety reduced (alongside improvements in well-being and resilience), even though the *Be Well Plan* was not developed to specifically address these outcomes directly, nor were participants provided with any traditional psychoeducational information about these mental health problems. This points to the program having value in both mental illness prevention and early intervention settings where current offerings are limited.

## Appendix

Multimedia Appendix 1 Weekly content of the 5-week Be Well Plan program.

Multimedia Appendix 2 CONSORT-eHEALTH checklist (V 1.6.1).

## Abbreviations

ACT: acceptance- and commitment-based therapy
CBT: cognitive behavior therapy
CD-RISC-10: 10-item Connor-Davidson Resilience Scale
CFS: Cognitive Flexibility Scale
GAD-7: 7-item General Anxiety Disorder
mITT: modified intention to treat
NGSES: New General Self-Efficacy Scale
PHQ-9: 9-item Patient Health Questionnaire
RCI: reliable change index
RCT: randomized controlled trial
SCS: Sense of Control Scale
WEMWBS: Warwick Edinburgh Mental Well-Being Scale
