# Supplementary material for: A Group-Facilitated, Internet-Based Intervention to Promote Mental Health and Well-Being in a Vulnerable Population of University Students: Randomized Controlled Trial of the Be Well Plan Program
Source: JMIR Ment Health. 2022 May 5;9(5):e37292. doi: 10.2196/37292 (PMC9084447; doi:10.2196/37292)
Supplement: Multimedia Appendix 1 [file mental_v9i5e37292_app1.docx]

**Multimedia Appendix 1. Weekly content of the 5-week Be Well Plan program.**

**Table S1.** Summary of 5 sessions of the *Be Well Plan*, as previously described in van Agteren et al [35,36].

| Session | Summary |
| --- | --- |
| **Session 1: getting on the same page** | Participants explore reasons for participating in the program, including their personal drivers. They also acquire *basic knowledge of mental health and well-being* and its malleability, which aims to stimulate a mindset for change. They continue by exploring the evidence for different psychological interventions and start developing their *Be Well Plan*. They do this by choosing a *mindfulness-based activity* and by setting a goal on how to practice this during the week. They get introduced to the formation of habit statements as a technique to improve the chance of goal attainment. |
| **Session 2: using your mental health profile** | Participants are introduced to the concept of *self-compassion* (as opposed to self-criticism) and how it can be used to learn from failure and shape our thinking patterns. After reflecting on session 1, they practice a self-compassion activity. They subsequently use their own mental health and well-being measurement results from the *Be Well Tracker* to focus on an outcome they want to work on (well-being, resilience, mood, anxiety, stress, or health) and are introduced to the activity finder, which allows them to select evidence-based activities that have been found to be effective in improving the outcomes mentioned before. They select 1 activity for their tailored *Be Well Plan* and are encouraged to set a new goal for the week. They are introduced to the use of *prompts and reminders* as another method to increase goal attainment. |
| **Session 3: your resources and challenges** | Participants learn about existing resources for their own mental health via 2 practical activities. The first encourages participants to choose pictures that display sources of *meaning in their life*, and the second asks participants to identify *core values* that can be used to guide their own goals. After reflecting on the previous session, participants then use a simple questionnaire to identify a key resource or challenge in their life they want to work on. They are introduced to a second activity finder, which maps evidence-based activities to various challenges and resources. They select a new activity from the activity bank to add to their *Be Well Plan*. They finish the session with reflecting on and adjusting their *Be Well Plan*. |
| **Session 4: stress, coping, and resilience** | This session focuses on stressful times and introduces *unhelpful and helpful coping strategies* (eg, avoidance-focused coping vs problem-focused coping). Participants learn a variety of different effective coping mechanisms as well as evidence-based skills from different therapeutic approaches (eg, CBT^a^, ACT^b^). After reflecting on the previous session, participants are asked to identify social supporters for challenging times and are reminded of various professional services. They are then encouraged to choose 1 new activity specifically focusing on stress and resilience. They are actively asked to reach out to a social supporter as part of their weekly activity in the *Be Well Plan*. |
| **Session 5: future-proofing your *Be Well Plan*** | Before the final session, participants are asked to complete a new measurement in the *Be Well Tracker* and investigate whether and how their outcomes have changed over the past 4 weeks. The facilitators introduce the concept of *realistic optimism* and discuss the fact that personal growth and progress come with ups and downs. After reflecting on the previous session, participants work on practicing positive reframing as a way to deal with mistakes and setbacks. They then build their final *Be Well Plan*, which aims to summarize key learnings from the previous weeks into a realistic mental health and well-being plan. They summarize what their best possible self would look like, highlight their unique drivers and motivations, and highlight existing resources and challenges in their life. They also set a longer-term goal and choose the activities they wish to add to their *Be Well Plan*. In addition, they identify their key supporters and reflect on what support services they need in the case of emergency. |

^a^CBT: cognitive behavior therapy.

^b^ACT: acceptance- and commitment-based therapy.
