# Supplementary material for: A Group-Facilitated, Internet-Based Intervention to Promote Mental Health and Well-Being in a Vulnerable Population of University Students: Randomized Controlled Trial of the Be Well Plan Program
Source: JMIR Ment Health. 2022 May 5;9(5):e37292. doi: 10.2196/37292 (PMC9084447; doi:10.2196/37292)
Supplement: Multimedia Appendix 2 [file mental_v9i5e37292_app2.pdf]

CONSORT-EHEALTH (V 1.6.1) - Submission/Publication Form

The CONSORT-EHEALTH checklist is intended for authors of randomized trials evaluating web-based and Internet-based applications/interventions, including mobile interventions, electronic games (incl multiplayer games), social media, certain telehealth applications, and other interactive and/or networked electronic applications. Some of the items (e.g. all subitems under item 5 - description of the intervention) may also be applicable for other study designs.

The goal of the CONSORT EHEALTH checklist and guideline is to be
a) a guide for reporting for authors of RCTs,
b) to form a basis for appraisal of an ehealth trial (in terms of validity)

CONSORT-EHEALTH items/subitems are MANDATORY reporting items for studies published in the Journal of Medical Internet Research and other journals / scientific societies endorsing the checklist.

Items numbered 1., 2., 3., 4a., 4b etc are original CONSORT or CONSORT-NPT (non-pharmacologic treatment) items.
Items with Roman numerals (i., ii, iii, iv etc.) are CONSORT-EHEALTH extensions/clarifications.

As the CONSORT-EHEALTH checklist is still considered in a formative stage, we would ask that you also RATE ON A SCALE OF 1-5 how important/useful you feel each item is FOR THE PURPOSE OF THE CHECKLIST and reporting guideline (optional).

Mandatory reporting items are marked with a red *.
In the textboxes, either copy & paste the relevant sections from your manuscript into this form - please include any quotes from your manuscript in QUOTATION MARKS,
or answer directly by providing additional information not in the manuscript, or elaborating on why the item was not relevant for this study.

YOUR ANSWERS WILL BE PUBLISHED AS A SUPPLEMENTARY FILE TO YOUR PUBLICATION IN JMIR AND ARE CONSIDERED PART OF YOUR PUBLICATION (IF ACCEPTED).
Please fill in these questions diligently. Information will not be copyedited, so please use proper spelling and grammar, use correct capitalization, and avoid abbreviations.

DO NOT FORGET TO SAVE AS PDF _AND_ CLICK THE SUBMIT BUTTON SO YOUR ANSWERS ARE IN OUR DATABASE !!!

Citation Suggestion (if you append the pdf as Appendix we suggest to cite this paper in the caption):
Eysenbach G, CONSORT-EHEALTH Group
CONSORT-EHEALTH: Improving and Standardizing Evaluation Reports of Web-based and Mobile Health Interventions

J Med Internet Res 2011;13(4):e126
URL: http://www.jmir.org/2011/4/e126/
doi: 10.2196/jmir.1923
PMID: 22209829

daniel.b.fassnacht@gmail.com wird nicht geteilt
Konto wechseln

Entwurf gespeichert

* Erforderlich

Your name *

First Last

Daniel

Primary Affiliation (short), City, Country *

University of Toronto, Toronto, Canada

Flinders University, Adelaide, Australia

Your e-mail address *

abc@gmail.com

dan.fassnacht@flinders.edu.au

Title of your manuscript *

Provide the (draft) title of your manuscript.

A facilitated, Internet-based intervention to promote mental health and wellbeing in a vulnerable population of university students: randomised controlled trial of the Be Well

Name of your App/Software/Intervention *

If there is a short and a long/alternate name, write the short name first and add the long name in brackets.

Be Well Plan

Evaluated Version (if any)

e.g. "V1", "Release 2017-03-01", "Version 2.0.27913"

Meine Antwort

Language(s) *

What language is the intervention/app in? If multiple languages are available, separate by comma (e.g. "English, French")

English

URL of your Intervention Website or App

e.g. a direct link to the mobile app on app in appstore (itunes, Google Play), or URL of the website. If the intervention is a DVD or hardware, you can also link to an Amazon page.

https://www.bewellco.io/bwp

URL of an image/screenshot (optional)

Meine Antwort

Accessibility *

Can an enduser access the intervention presently?

- ○ access is free and open
- ○ access only for special usergroups, not open
- ◉ access is open to everyone, but requires payment/subscription/in-app purchases
- ○ app/intervention no longer accessible
- ○ Sonstiges:

Primary Medical Indication/Disease/Condition *

e.g. "Stress", "Diabetes", or define the target group in brackets after the condition, e.g. "Autism (Parents of children with)", "Alzheimers (Informal Caregivers of)"

Mental wellbeing, psychological distress

Primary Outcomes measured in trial *

comma-separated list of primary outcomes reported in the trial

Wellbeing, resilience, depression, anxiety

Secondary/other outcomes

Are there any other outcomes the intervention is expected to affect?

Self-efficacy, sense of control, cognitive flexibility

Recommended "Dose" *

What do the instructions for users say on how often the app should be used?

- ( ) Approximately Daily
- (x) Approximately Weekly
- ( ) Approximately Monthly
- ( ) Approximately Yearly
- ( ) "as needed"
- ( ) Sonstiges:

Approx. Percentage of Users (starters) still using the app as recommended after 3 months *

- ( ) unknown / not evaluated
- ( ) 0-10%
- ( ) 11-20%
- ( ) 21-30%
- ( ) 31-40%
- ( ) 41-50%
- ( ) 51-60%
- ( ) 61-70%
- ( ) 71%-80%
- ( ) 81-90%
- ( ) 91-100%
- (x) Sonstiges: N/A

Overall, was the app/intervention effective? *

- (x) yes: all primary outcomes were significantly better in intervention group vs control
- ( ) partly: SOME primary outcomes were significantly better in intervention group vs control
- ( ) no statistically significant difference between control and intervention
- ( ) potentially harmful: control was significantly better than intervention in one or more outcomes
- ( ) inconclusive: more research is needed
- ( ) Sonstiges:

Article Preparation Status/Stage *

At which stage in your article preparation are you currently (at the time you fill in this form)

- ( ) not submitted yet - in early draft status
- (x) not submitted yet - in late draft status, just before submission
- ( ) submitted to a journal but not reviewed yet
- ( ) submitted to a journal and after receiving initial reviewer comments
- ( ) submitted to a journal and accepted, but not published yet
- ( ) published
- ( ) Sonstiges:

Journal *

If you already know where you will submit this paper (or if it is already submitted), please provide the journal name (if it is not JMIR, provide the journal name under "other")

- ○ not submitted yet / unclear where I will submit this
- ◉ Journal of Medical Internet Research (JMIR)
- ○ JMIR mHealth and UHealth
- ○ JMIR Serious Games
- ○ JMIR Mental Health
- ○ JMIR Public Health
- ○ JMIR Formative Research
- ○ Other JMIR sister journal
- ○ Sonstiges:

Is this a full powered effectiveness trial or a pilot/feasibility trial? *

- ○ Pilot/feasibility
- ◉ Fully powered

Manuscript tracking number *

If this is a JMIR submission, please provide the manuscript tracking number under "other" (The ms tracking number can be found in the submission acknowledgement email, or when you login as author in JMIR. If the paper is already published in JMIR, then the ms tracking number is the four-digit number at the end of the DOI, to be found at the bottom of each published article in JMIR)

- ◉ no ms number (yet) / not (yet) submitted to / published in JMIR
- ○ Sonstiges:

TITLE AND ABSTRACT

1a) TITLE: Identification as a randomized trial in the title

1a) Does your paper address CONSORT item 1a? *

I.e does the title contain the phrase "Randomized Controlled Trial"? (if not, explain the reason under "other")

- (x) yes
- ( ) Sonstiges:

1a-i) Identify the mode of delivery in the title

Identify the mode of delivery. Preferably use “web-based” and/or “mobile” and/or “electronic game” in the title. Avoid ambiguous terms like “online”, “virtual”, “interactive”. Use “Internet-based” only if Intervention includes non-web-based Internet components (e.g. email), use “computer-based” or “electronic” only if offline products are used. Use “virtual” only in the context of “virtual reality” (3-D worlds). Use “online” only in the context of “online support groups”. Complement or substitute product names with broader terms for the class of products (such as “mobile” or “smart phone” instead of “iphone”), especially if the application runs on different platforms.

	1	2	3	4	5	
subitem not at all important	( )	( )	( )	( )	( )	essential

Does your paper address subitem 1a-i? *

Copy and paste relevant sections from manuscript title (include quotes in quotation marks "like this" to indicate direct quotes from your manuscript), or elaborate on this item by providing additional information not in the ms, or briefly explain why the item is not applicable/relevant for your study

"A facilitated, Internet-based intervention..."
"The Be Well Plan, a 5-week facilitated, Internet-based mental health and wellbeing group intervention..."

1a-ii) Non-web-based components or important co-interventions in title

Mention non-web-based components or important co-interventions in title, if any (e.g., "with telephone support").

	1	2	3	4	5	
subitem not at all important	○	○	○	○	○	essential

Does your paper address subitem 1a-ii?

Copy and paste relevant sections from manuscript title (include quotes in quotation marks "like this" to indicate direct quotes from your manuscript), or elaborate on this item by providing additional information not in the ms, or briefly explain why the item is not applicable/relevant for your study

Meine Antwort

1a-iii) Primary condition or target group in the title

Mention primary condition or target group in the title, if any (e.g., "for children with Type I Diabetes") Example: A Web-based and Mobile Intervention with Telephone Support for Children with Type I Diabetes: Randomized Controlled Trial

	1	2	3	4	5	
subitem not at all important	○	○	○	○	○	essential

Does your paper address subitem 1a-iii? *

Copy and paste relevant sections from manuscript title (include quotes in quotation marks "like this" to indicate direct quotes from your manuscript), or elaborate on this item by providing additional information not in the ms, or briefly explain why the item is not applicable/relevant for your study

"...in a vulnerable population of university students."

1b) ABSTRACT: Structured summary of trial design, methods, results, and conclusions

NPT extension: Description of experimental treatment, comparator, care providers, centers, and blinding status.

1b-i) Key features/functionalities/components of the intervention and comparator in the METHODS section of the ABSTRACT

Mention key features/functionalities/components of the intervention and comparator in the abstract. If possible, also mention theories and principles used for designing the site. Keep in mind the needs of systematic reviewers and indexers by including important synonyms. (Note: Only report in the abstract what the main paper is reporting. If this information is missing from the main body of text, consider adding it)

	1	2	3	4	5	
subitem not at all important	○	○	○	○	○	essential

Does your paper address subitem 1b-i? *

Copy and paste relevant sections from the manuscript abstract (include quotes in quotation marks "like this" to indicate direct quotes from your manuscript), or elaborate on this item by providing additional information not in the ms, or briefly explain why the item is not applicable/relevant for your study

"A randomised controlled trial was conducted with two parallel arms, an intervention and a wait-list control group. "

"The intervention introduces participants to a wide range of evidence-based psychological activities (e.g., mindfulness, problem solving, self-compassion, thought challenging)..."

"Over a period of five weeks, participants are introduced to different methods to select and experiment with activities that are relevant to their unique needs."

"...the program was designed to be delivered using a train-the-trainer methodology. Facilitators have undergone a structured training schedule before they guide participants through the program."

1b-ii) Level of human involvement in the METHODS section of the ABSTRACT

Clarify the level of human involvement in the abstract, e.g., use phrases like “fully automated” vs. “therapist/nurse/care provider/physician-assisted” (mention number and expertise of providers involved, if any). (Note: Only report in the abstract what the main paper is reporting. If this information is missing from the main body of text, consider adding it)

	1	2	3	4	5	
subitem not at all important	○	○	○	○	○	essential

Does your paper address subitem 1b-ii?

Copy and paste relevant sections from the manuscript abstract (include quotes in quotation marks "like this" to indicate direct quotes from your manuscript), or elaborate on this item by providing additional information not in the ms, or briefly explain why the item is not applicable/relevant for your study

Meine Antwort

1b-iii) Open vs. closed, web-based (self-assessment) vs. face-to-face assessments in the METHODS section of the ABSTRACT

Mention how participants were recruited (online vs. offline), e.g., from an open access website or from a clinic or a closed online user group (closed usergroup trial), and clarify if this was a purely web-based trial, or there were face-to-face components (as part of the intervention or for assessment). Clearly say if outcomes were self-assessed through questionnaires (as common in web-based trials). Note: In traditional offline trials, an open trial (open-label trial) is a type of clinical trial in which both the researchers and participants know which treatment is being administered. To avoid confusion, use “blinded” or “unblinded” to indicated the level of blinding instead of “open”, as “open” in web-based trials usually refers to “open access” (i.e. participants can self-enrol). (Note: Only report in the abstract what the main paper is reporting. If this information is missing from the main body of text, consider adding it)

	1	2	3	4	5	
subitem not at all important	○	○	○	○	○	essential

Does your paper address subitem 1b-iii?

Copy and paste relevant sections from the manuscript abstract (include quotes in quotation marks "like this" to indicate direct quotes from your manuscript), or elaborate on this item by providing additional information not in the ms, or briefly explain why the item is not applicable/relevant for your study

Meine Antwort

1b-iv) RESULTS section in abstract must contain use data

Report number of participants enrolled/assessed in each group, the use/uptake of the intervention (e.g., attrition/adherence metrics, use over time, number of logins etc.), in addition to primary/secondary outcomes. (Note: Only report in the abstract what the main paper is reporting. If this information is missing from the main body of text, consider adding it)

	1	2	3	4	5	
subitem not at all important	○	○	○	○	○	essential

Does your paper address subitem 1b-iv?

Copy and paste relevant sections from the manuscript abstract (include quotes in quotation marks "like this" to indicate direct quotes from your manuscript), or elaborate on this item by providing additional information not in the ms, or briefly explain why the item is not applicable/relevant for your study

Meine Antwort

1b-v) CONCLUSIONS/DISCUSSION in abstract for negative trials

Conclusions/Discussions in abstract for negative trials: Discuss the primary outcome - if the trial is negative (primary outcome not changed), and the intervention was not used, discuss whether negative results are attributable to lack of uptake and discuss reasons. (Note: Only report in the abstract what the main paper is reporting. If this information is missing from the main body of text, consider adding it)

	1	2	3	4	5	
subitem not at all important	○	○	○	○	○	essential

Does your paper address subitem 1b-v?

Copy and paste relevant sections from the manuscript abstract (include quotes in quotation marks "like this" to indicate direct quotes from your manuscript), or elaborate on this item by providing additional information not in the ms, or briefly explain why the item is not applicable/relevant for your study

Meine Antwort

INTRODUCTION

2a) In INTRODUCTION: Scientific background and explanation of rationale

2a-i) Problem and the type of system/solution

Describe the problem and the type of system/solution that is object of the study: intended as stand-alone intervention vs. incorporated in broader health care program? Intended for a particular patient population? Goals of the intervention, e.g., being more cost-effective to other interventions, replace or complement other solutions? (Note: Details about the intervention are provided in “Methods” under 5)

	1	2	3	4	5	
subitem not at all important	○	○	○	○	○	essential

Does your paper address subitem 2a-i? *

Copy and paste relevant sections from the manuscript (include quotes in quotation marks "like this" to indicate direct quotes from your manuscript), or elaborate on this item by providing additional information not in the ms, or briefly explain why the item is not applicable/relevant for your study

"As such, our team has developed an intervention that explicitly targets both mental health and wellbeing, the “Be Well Plan”"

"In contrast to more structured and manualised interventions which often provide a set number of activities as part of a fixed program, participants in the Be Well Plan can choose from an activity bank with more than 30 different activities. Throughout the program participants are supported in experimenting with different activities while learning which of them work well for them as individuals with their specific needs and preferences."

"In addition, the Be Well Plan can successfully reach vulnerable populations, who may be isolated or struggling, but are not ready or able to access contemporary services due to

2a-ii) Scientific background, rationale: What is known about the (type of) system

Scientific background, rationale: What is known about the (type of) system that is the object of the study (be sure to discuss the use of similar systems for other conditions/diagnoses, if appropiate), motivation for the study, i.e. what are the reasons for and what is the context for this specific study, from which stakeholder viewpoint is the study performed, potential impact of findings [2]. Briefly justify the choice of the comparator.

	1	2	3	4	5	
subitem not at all important	○	○	○	○	○	essential

Does your paper address subitem 2a-ii? *

Copy and paste relevant sections from the manuscript (include quotes in quotation marks "like this" to indicate direct quotes from your manuscript), or elaborate on this item by providing additional information not in the ms, or briefly explain why the item is not applicable/relevant for your study

"While stepped models of care theoretically include a focus on building or promoting good mental health, the models are conceptually designed to deal with the impact of illness. As such, the solutions implemented across the continuum focus on preventing or treating symptoms of illness, not necessarily promoting good mental health (Keyes, 2007; Richards et al., 2012)."

"For example, chemo- and radiotherapy helps to treat cancer but generally is not considered to be helpful for improving overall health status (Baskar et al., 2012; Schirrmacher, 2019). In contrast, good nutrition and physical activity does improve overall health and may aid in the recovery process from cancer but is generally not sufficient to stop established cancer from advancing on its own. There is a clearly overlooked opportunity for mental healthcare to mirror this parallel and to systematically adopt solutions with a broader focus than simply targeting or aiming to prevent and treat symptoms of mental illness. In other words, instead of an abundant reliance on reactive solutions there may be a place for pro-active solutions that promote mental health and

2b) In INTRODUCTION: Specific objectives or hypotheses

Does your paper address CONSORT subitem 2b? *

Copy and paste relevant sections from the manuscript (include quotes in quotation marks "like this" to indicate direct quotes from your manuscript), or elaborate on this item by providing additional information not in the ms, or briefly explain why the item is not applicable/relevant for your study

"While preliminary evidence for the Be Well Plan's impact on mental health and wellbeing has been established (Van Agteren, Ali, et al., 2021b), there is a clear need to investigate its efficacy with a more robust methodological design using a randomised controlled trial. This study aimed to advance the literature in several ways. First, we aimed to test the efficacy of the Be Well Plan with a vulnerable population (university students) in improving primary outcomes of mental wellbeing, resilience, anxiety and depression and secondary outcomes of self-efficacy, sense of control, and cognitive flexibility. Second, we aimed to examine participants' engagement and satisfaction with the Be Well Plan facilitated in a group format using teleconferencing software."

METHODS

3a) Description of trial design (such as parallel, factorial) including allocation ratio

Does your paper address CONSORT subitem 3a? *

Copy and paste relevant sections from the manuscript (include quotes in quotation marks "like this" to indicate direct quotes from your manuscript), or elaborate on this item by providing additional information not in the ms, or briefly explain why the item is not applicable/relevant for your study

"A two-arm RCT was conducted comparing an active intervention (Be Well Plan) with a wait-list control condition."

3b) Important changes to methods after trial commencement (such as eligibility criteria), with reasons

Does your paper address CONSORT subitem 3b? *

Copy and paste relevant sections from the manuscript (include quotes in quotation marks "like this" to indicate direct quotes from your manuscript), or elaborate on this item by providing additional information not in the ms, or briefly explain why the item is not applicable/relevant for your study

There were no changes after trial commencement; see also trial registration.

"The trial was ... registered with the Australian New Zealand Clinical Trial Registry (ACTRN12621000180819)."

3b-i) Bug fixes, Downtimes, Content Changes

Bug fixes, Downtimes, Content Changes: ehealth systems are often dynamic systems. A description of changes to methods therefore also includes important changes made on the intervention or comparator during the trial (e.g., major bug fixes or changes in the functionality or content) (5-iii) and other “unexpected events” that may have influenced study design such as staff changes, system failures/downtimes, etc. [2].

	1	2	3	4	5	
subitem not at all important	○	○	○	○	○	essential

Does your paper address subitem 3b-i?

Copy and paste relevant sections from the manuscript (include quotes in quotation marks "like this" to indicate direct quotes from your manuscript), or elaborate on this item by providing additional information not in the ms, or briefly explain why the item is not applicable/relevant for your study

Meine Antwort

4a) Eligibility criteria for participants

Does your paper address CONSORT subitem 4a? *

Copy and paste relevant sections from the manuscript (include quotes in quotation marks "like this" to indicate direct quotes from your manuscript), or elaborate on this item by providing additional information not in the ms, or briefly explain why the item is not applicable/relevant for your study

"University students were recruited between August 2020 and April 2021 through emails, lectures, social media posts, posters and flyers at a medium-size public university (approx. 24,500 enrolled students in 2020) in Adelaide, Australia."

"All enrolled students across the university were eligible to participate; no other eligibility criteria applied. ... English language proficiency was assumed, as all students had passed the university language requirement for English before their university enrolment. Similarly, given the requirements of tertiary study, computer and internet literacy was assumed."

4a-i) Computer / Internet literacy

Computer / Internet literacy is often an implicit "de facto" eligibility criterion - this should be explicitly clarified.

	1	2	3	4	5	
subitem not at all important	○	○	○	○	○	essential

Does your paper address subitem 4a-i?

Copy and paste relevant sections from the manuscript (include quotes in quotation marks "like this" to indicate direct quotes from your manuscript), or elaborate on this item by providing additional information not in the ms, or briefly explain why the item is not applicable/relevant for your study

Meine Antwort

4a-ii) Open vs. closed, web-based vs. face-to-face assessments:

Open vs. closed, web-based vs. face-to-face assessments: Mention how participants were recruited (online vs. offline), e.g., from an open access website or from a clinic, and clarify if this was a purely web-based trial, or there were face-to-face components (as part of the intervention or for assessment), i.e., to what degree got the study team to know the participant. In online-only trials, clarify if participants were quasi-anonymous and whether having multiple identities was possible or whether technical or logistical measures (e.g., cookies, email confirmation, phone calls) were used to detect/prevent these.

	1	2	3	4	5	
subitem not at all important	○	○	○	○	○	essential

Does your paper address subitem 4a-ii? *

Copy and paste relevant sections from the manuscript (include quotes in quotation marks "like this" to indicate direct quotes from your manuscript), or elaborate on this item by providing additional information not in the ms, or briefly explain why the item is not applicable/relevant for your study

"University students were recruited between August 2020 and April 2021 through emails, lectures, social media posts, posters and flyers at a medium-size public university (approx. 24,500 enrolled students in 2020) in Adelaide, Australia. Recruitment messaging focused on inviting students to participate in a new program that aimed to build their mental health and wellbeing."

"Participants registered their interest via an online survey and indicated their preferred day and time for the five intervention sessions. They could then attend a general information session about the content and structure of the program, hosted online via the teleconferencing software Zoom, or watch a pre-recorded version. After providing their informed consent electronically to participate in the trial, participants completed the baseline assessment online including primary (i.e., wellbeing, resilience, depression, anxiety) and secondary outcome measures (self-efficacy, perceived sense of control, cognitive flexibility) as well as general demographic questions (i.e., age, gender, ethnicity, student and employment status) and their overall health status (i.e., diet, activity, sleep)."

4a-iii) Information giving during recruitment

Information given during recruitment. Specify how participants were briefed for recruitment and in the informed consent procedures (e.g., publish the informed consent documentation as appendix, see also item X26), as this information may have an effect on user self-selection, user expectation and may also bias results.

	1	2	3	4	5	
subitem not at all important	○	○	○	○	○	essential

Does your paper address subitem 4a-iii?

Copy and paste relevant sections from the manuscript (include quotes in quotation marks "like this" to indicate direct quotes from your manuscript), or elaborate on this item by providing additional information not in the ms, or briefly explain why the item is not applicable/relevant for your study

Meine Antwort

4b) Settings and locations where the data were collected

Does your paper address CONSORT subitem 4b? *

Copy and paste relevant sections from the manuscript (include quotes in quotation marks "like this" to indicate direct quotes from your manuscript), or elaborate on this item by providing additional information not in the ms, or briefly explain why the item is not applicable/relevant for your study

"After providing their informed consent electronically to participate in the trial, participants completed the baseline assessment online including primary (i.e., wellbeing, resilience, depression, anxiety) and secondary outcome measures (self-efficacy, perceived sense of control, cognitive flexibility) as well as general demographic questions (i.e., age, gender, ethnicity, student and employment status) and their overall health status (i.e., diet, activity, sleep)."

4b-i) Report if outcomes were (self-)assessed through online questionnaires

Clearly report if outcomes were (self-)assessed through online questionnaires (as common in web-based trials) or otherwise.

	1	2	3	4	5	
subitem not at all important	○	○	○	○	○	essential

Does your paper address subitem 4b-i? *

Copy and paste relevant sections from the manuscript (include quotes in quotation marks "like this" to indicate direct quotes from your manuscript), or elaborate on this item by providing additional information not in the ms, or briefly explain why the item is not applicable/relevant for your study

"After providing their informed consent electronically to participate in the trial, participants completed the baseline assessment online including primary (i.e., wellbeing, resilience, depression, anxiety) and secondary outcome measures (self-efficacy, perceived sense of control, cognitive flexibility) as well as general demographic questions (i.e., age, gender, ethnicity, student and employment status) and their overall health status (i.e., diet, activity, sleep)."

4b-ii) Report how institutional affiliations are displayed

Report how institutional affiliations are displayed to potential participants [on ehealth media], as affiliations with prestigious hospitals or universities may affect volunteer rates, use, and reactions with regards to an intervention.(Not a required item – describe only if this may bias results)

	1	2	3	4	5	
subitem not at all important	○	○	○	○	○	essential

Does your paper address subitem 4b-ii?

Copy and paste relevant sections from the manuscript (include quotes in quotation marks "like this" to indicate direct quotes from your manuscript), or elaborate on this item by providing additional information not in the ms, or briefly explain why the item is not applicable/relevant for your study

Meine Antwort

5) The interventions for each group with sufficient details to allow replication, including how and when they were actually administered

5-i) Mention names, credential, affiliations of the developers, sponsors, and owners

Mention names, credential, affiliations of the developers, sponsors, and owners [6] (if authors/evaluators are owners or developer of the software, this needs to be declared in a “Conflict of interest” section or mentioned elsewhere in the manuscript).

	1	2	3	4	5	
subitem not at all important	○	○	○	○	○	essential

Does your paper address subitem 5-i?

Copy and paste relevant sections from the manuscript (include quotes in quotation marks "like this" to indicate direct quotes from your manuscript), or elaborate on this item by providing additional information not in the ms, or briefly explain why the item is not applicable/relevant for your study

Meine Antwort

5-ii) Describe the history/development process

Describe the history/development process of the application and previous formative evaluations (e.g., focus groups, usability testing), as these will have an impact on adoption/use rates and help with interpreting results.

	1	2	3	4	5	
subitem not at all important	○	○	○	○	○	essential

Does your paper address subitem 5-ii?

Copy and paste relevant sections from the manuscript (include quotes in quotation marks "like this" to indicate direct quotes from your manuscript), or elaborate on this item by providing additional information not in the ms, or briefly explain why the item is not applicable/relevant for your study

Meine Antwort

5-iii) Revisions and updating

Revisions and updating. Clearly mention the date and/or version number of the application/intervention (and comparator, if applicable) evaluated, or describe whether the intervention underwent major changes during the evaluation process, or whether the development and/or content was “frozen” during the trial. Describe dynamic components such as news feeds or changing content which may have an impact on the replicability of the intervention (for unexpected events see item 3b).

	1	2	3	4	5	
subitem not at all important	○	○	○	○	○	essential

Does your paper address subitem 5-iii?

Copy and paste relevant sections from the manuscript (include quotes in quotation marks "like this" to indicate direct quotes from your manuscript), or elaborate on this item by providing additional information not in the ms, or briefly explain why the item is not applicable/relevant for your study

Meine Antwort

5-iv) Quality assurance methods

Provide information on quality assurance methods to ensure accuracy and quality of information provided [1], if applicable.

	1	2	3	4	5	
subitem not at all important	○	○	○	○	○	essential

Does your paper address subitem 5-iv?

Copy and paste relevant sections from the manuscript (include quotes in quotation marks "like this" to indicate direct quotes from your manuscript), or elaborate on this item by providing additional information not in the ms, or briefly explain why the item is not applicable/relevant for your study

Meine Antwort

5-v) Ensure replicability by publishing the source code, and/or providing screenshots/screen-capture video, and/or providing flowcharts of the algorithms used

Ensure replicability by publishing the source code, and/or providing screenshots/screen-capture video, and/or providing flowcharts of the algorithms used. Replicability (i.e., other researchers should in principle be able to replicate the study) is a hallmark of scientific reporting.

	1	2	3	4	5	
subitem not at all important	○	○	○	○	○	essential

Does your paper address subitem 5-v?

Copy and paste relevant sections from the manuscript (include quotes in quotation marks "like this" to indicate direct quotes from your manuscript), or elaborate on this item by providing additional information not in the ms, or briefly explain why the item is not applicable/relevant for your study

Meine Antwort

5-vi) Digital preservation

Digital preservation: Provide the URL of the application, but as the intervention is likely to change or disappear over the course of the years; also make sure the intervention is archived (Internet Archive, webcitation.org, and/or publishing the source code or screenshots/videos alongside the article). As pages behind login screens cannot be archived, consider creating demo pages which are accessible without login.

	1	2	3	4	5	
subitem not at all important	○	○	○	○	○	essential

Does your paper address subitem 5-vi?

Copy and paste relevant sections from the manuscript (include quotes in quotation marks "like this" to indicate direct quotes from your manuscript), or elaborate on this item by providing additional information not in the ms, or briefly explain why the item is not applicable/relevant for your study

Meine Antwort

5-vii) Access

Access: Describe how participants accessed the application, in what setting/context, if they had to pay (or were paid) or not, whether they had to be a member of specific group. If known, describe how participants obtained “access to the platform and Internet” [1]. To ensure access for editors/reviewers /readers, consider to provide a “backdoor” login account or demo mode for reviewers/readers to explore the application (also important for archiving purposes, see vi).

	1	2	3	4	5	
subitem not at all important	○	○	○	○	○	essential

Does your paper address subitem 5-vii? *

Copy and paste relevant sections from the manuscript (include quotes in quotation marks "like this" to indicate direct quotes from your manuscript), or elaborate on this item by providing additional information not in the ms, or briefly explain why the item is not applicable/relevant for your study

"Participants allocated to the intervention group received detailed information including the Zoom link to the first session of the Be Well Plan prior to the start of the program. They further received a separate email inviting them to complete a brief 10-to-15-minute survey to assess their levels of mental health and wellbeing via a platform called the Be Well Tracker. After completing the Be Well Tracker, participants received a detailed report about their levels of wellbeing, resilience, and distress which provided them with relevant information that would be used throughout the Be Well Plan intervention."

5-viii) Mode of delivery, features/functionalities/components of the intervention and comparator, and the theoretical framework

Describe mode of delivery, features/functionalities/components of the intervention and comparator, and the theoretical framework [6] used to design them (instructional strategy [1], behaviour change techniques, persuasive features, etc., see e.g., [7, 8] for terminology). This includes an in-depth description of the content (including where it is coming from and who developed it) [1],” whether [and how] it is tailored to individual circumstances and allows users to track their progress and receive feedback” [6]. This also includes a description of communication delivery channels and – if computer-mediated communication is a component – whether communication was synchronous or asynchronous [6]. It also includes information on presentation strategies [1], including page design principles, average amount of text on pages, presence of hyperlinks to other resources, etc. [1].

	1	2	3	4	5	
subitem not at all important	○	○	○	○	○	essential

Does your paper address subitem 5-viii? *

Copy and paste relevant sections from the manuscript (include quotes in quotation marks "like this" to indicate direct quotes from your manuscript), or elaborate on this item by providing additional information not in the ms, or briefly explain why the item is not applicable/relevant for your study

"The Be Well Plan intervention has been previously described in a detailed manuscript by van Agteren, Iasiello, Ali, et al. (2021b), outlining the individual components of the program and providing insights into program materials, including screenshots of the intervention. In summary, the Be Well Plan is a weekly, 5-session Internet-based facilitated group intervention which aims to improve mental health and wellbeing. The program assists participants in developing their own wellbeing plan tailored to their individual circumstances and needs. Participants learn and experiment with a range of evidence-based activities and skills targeted at improving mental health and wellbeing. Each session provides evidence-based information, self-reflection activities and sharing of experiences between participants. The Be Well Plan introduces participants to an activity bank consisting of more than 30 evidence-based activities which were selected from a large meta-analysis and assists participants in identifying those which would likely work for their particular needs and circumstances (van Agteren, Iasiello, Lo, et al., 2021b). Each session was conducted via Zoom by two trained facilitators to ensure that the program adhered to the intervention protocol (van Agteren, Iasiello, Ali, et al., 2021a) and was delivered in an engaging and safe way. In total, five facilitators (KA, JVA, MI, TM, GF, AH, KS) with a variety of professional backgrounds including wellbeing research, counselling, and clinical psychology delivered the program."

"Participants in the wait-list control group were asked to complete the pre-and post-intervention assessment after which they were provided with access to the 5-week facilitated Be Well Plan sessions."

5-ix) Describe use parameters

Describe use parameters (e.g., intended “doses” and optimal timing for use). Clarify what instructions or recommendations were given to the user, e.g., regarding timing, frequency, heaviness of use, if any, or was the intervention used ad libitum.

	1	2	3	4	5	
subitem not at all important	○	○	○	○	○	essential

Does your paper address subitem 5-ix?

Copy and paste relevant sections from the manuscript (include quotes in quotation marks "like this" to indicate direct quotes from your manuscript), or elaborate on this item by providing additional information not in the ms, or briefly explain why the item is not applicable/relevant for your study

Meine Antwort

5-x) Clarify the level of human involvement

Clarify the level of human involvement (care providers or health professionals, also technical assistance) in the e-intervention or as co-intervention (detail number and expertise of professionals involved, if any, as well as “type of assistance offered, the timing and frequency of the support, how it is initiated, and the medium by which the assistance is delivered”. It may be necessary to distinguish between the level of human involvement required for the trial, and the level of human involvement required for a routine application outside of a RCT setting (discuss under item 21 – generalizability).

	1	2	3	4	5	
subitem not at all important	○	○	○	○	○	essential

Does your paper address subitem 5-x?

Copy and paste relevant sections from the manuscript (include quotes in quotation marks "like this" to indicate direct quotes from your manuscript), or elaborate on this item by providing additional information not in the ms, or briefly explain why the item is not applicable/relevant for your study

Meine Antwort

5-xi) Report any prompts/reminders used

Report any prompts/reminders used: Clarify if there were prompts (letters, emails, phone calls, SMS) to use the application, what triggered them, frequency etc. It may be necessary to distinguish between the level of prompts/reminders required for the trial, and the level of prompts/reminders for a routine application outside of a RCT setting (discuss under item 21 – generalizability).

	1	2	3	4	5	
subitem not at all important	○	○	○	○	○	essential

Does your paper address subitem 5-xi? *

Copy and paste relevant sections from the manuscript (include quotes in quotation marks "like this" to indicate direct quotes from your manuscript), or elaborate on this item by providing additional information not in the ms, or briefly explain why the item is not applicable/relevant for your study

"Up to three email reminders were sent to participants to complete the assessment."

5-xii) Describe any co-interventions (incl. training/support)

Describe any co-interventions (incl. training/support): Clearly state any interventions that are provided in addition to the targeted eHealth intervention, as ehealth intervention may not be designed as stand-alone intervention. This includes training sessions and support [1]. It may be necessary to distinguish between the level of training required for the trial, and the level of training for a routine application outside of a RCT setting (discuss under item 21 – generalizability.

	1	2	3	4	5	
subitem not at all important	○	○	○	○	○	essential

Does your paper address subitem 5-xii? *

Copy and paste relevant sections from the manuscript (include quotes in quotation marks "like this" to indicate direct quotes from your manuscript), or elaborate on this item by providing additional information not in the ms, or briefly explain why the item is not applicable/relevant for your study

"A key feature of the Be Well Plan is that the program does not rely on mental health trained clinicians. Instead, the program was designed to be delivered using a train-the-trainer methodology. Facilitators have undergone a structured training schedule before they guide participants through the program."

6a) Completely defined pre-specified primary and secondary outcome measures, including how and when they were assessed

Does your paper address CONSORT subitem 6a? *

Copy and paste relevant sections from the manuscript (include quotes in quotation marks "like this" to indicate direct quotes from your manuscript), or elaborate on this item by providing additional information not in the ms, or briefly explain why the item is not applicable/relevant for your study

"After providing their informed consent electronically to participate in the trial, participants completed the baseline assessment online including primary (i.e., wellbeing, resilience, depression, anxiety) and secondary outcome measures (self-efficacy, perceived sense of control, cognitive flexibility) as well as general demographic questions (i.e., age, gender, ethnicity, student and employment status) and their overall health status (i.e., diet, activity, sleep)."

"Six weeks after the baseline assessment, participants in both groups were asked to complete another online assessment including the primary and secondary outcome measures. This meant participants from the intervention condition completed the post-intervention assessment one week after the final session of the program. Participants from the intervention condition were also asked questions about their satisfaction with the program. Participants from the wait-list condition were given access to the intervention following their second assessment. Up to three email reminders were sent to participants to complete the assessment."

"Outcome Measures
Primary outcome measures assessed participants' wellbeing and resilience as well as their levels of depressive and anxiety symptoms. Secondary outcomes included self-efficacy, sense of control and cognitive flexibility.
Wellbeing. The Warwick Edinburgh Mental Well-Being Scale (WEMWBS) was used to assess mental wellbeing including eudaimonic and hedonic aspects of wellbeing (Tennant et al., 2007). The 14-item scale asks participants to indicate how often, over the past two weeks, from 0 (none of the time) to 5 (all of the time) they have experienced different thoughts and feelings (e.g., "I've been feeling useful."). Total scores range from 0 to 70, with higher scores indicating greater levels of mental wellbeing. Tennant et al. (2007) found that the WEMWBS demonstrated good content and construct validity, adequate test-retest reliability, and good internal consistency (Cronbach α = .83). The internal consistency of the WEMWBS in the current study was excellent (Cronbach's α = .91).
Resilience. The 10-item Connor-Davidson Resilience Scale-10 (CD-RISC-10) (Campbell-Sills et al., 2009) was used to assess resilience. Participants respond on a 5-point Likert scale from 0 (not true at all) to 4 (true nearly all the time) on how well they cope with adversity (e.g., "I am able to adapt when changes occur"). Total scores range from 0 to 40, with higher scores indicating greater levels of resilience. The CD-RISC-10 has demonstrated good construct validity and internal consistency (Cronbach's α = .85) (Campbell-Sills & Stein, 2007). The internal consistency of the CDRS in the current study was good (Cronbach's α = .88).
Depression. The 9-item Patient Health Questionnaire-9 (PHQ-9) (Kroenke et al., 2001) was used to assess symptoms of depression. Participants respond on a 4-point Likert scale from 0 (not at all) to 3 (nearly every day) how often they have experienced depressive symptoms (e.g., "feeling tired or having little energy" or "feeling down, depressed, or hopeless") over the last two weeks. Total scores range from 0 to 27, with higher scores indicating greater levels of depressive symptoms. The PHQ-9 has demonstrated good construct validity and internal consistency (Cronbach's α = .86 – .89) (Kroenke et al., 2001). The internal consistency of the PHQ-9 in the current sample was good (Cronbach's α = .85). The following cut-offs were used in the current study: 0–9 = no to mild depressive symptoms, ≥10 = moderate to severe depressive symptoms (Kroenke et al., 2001).
Anxiety. The 7-item General Anxiety Disorder-7 (GAD-7) (Spitzer et al., 2006) was used to assess symptoms of anxiety. Participants respond on a 4-point Likert scale from 0 (not at all) to 3 (nearly every day) how often they have experienced symptoms of anxiety (e.g., "feeling nervous, anxious or on edge" or "trouble relaxing") over the last two weeks. Total

[illegible] scores range from 0 to 21, with higher scores indicating greater levels of anxiety. The GAD-7 has demonstrated good construct validity and internal consistency (Cronbach's α = .91) (Spitzer et al., 2006). The internal consistency of the GAD-7 in the current sample was good (Cronbach's α = .85). The following cut-offs were used in the current study: 0–9 = minimal to mild anxiety, ≥ 10 = moderate to severe anxiety (Spitzer et al., 2006).
Self-efficacy. The 8-item New General Self-Efficacy Scale (NGSES) (Chen et al., 2001) was used to assess levels of general self-efficacy. Participants respond on a 5-point Likert scale from 1 (strongly disagree) to 5 (strongly agree) how often they believe they can achieve their goals, despite difficulties (e.g., "When facing difficult tasks, I am certain that I will accomplish them."). Total scores range from 1 to 5, with higher scores indicating greater levels of self-efficacy. The NGSES demonstrated good predictive validity and internal consistency (Cronbach's α = .85 – .91) (Chen et al., 2001). The internal consistency of the NGSES in the current sample was good (Cronbach's α = .87).
Sense of Control. The 12-item Sense of Control Scale (SCS) (Lachman & Weaver, 1998) was used to assess participants' perceived sense of control over their lives. Participants respond on a 7-point Likert scale from 1 (strongly agree) to 7 (strongly disagree) how much they feel they can control (e.g., "Whether or not I am able to get what I want is in my own hands") or not control (e.g., "Other people determine most of what I can and cannot do") their personal lives. Total scores range from 1 to 7, with higher scores indicating greater control. The internal consistency of the SCS in the current sample was good (Cronbach's α = .84).
Cognitive Flexibility. The 12-item Cognitive Flexibility Scale (CFS) (Martin & Rubin, 1995) was used to assess participants' mental and cognitive flexibility. Participants respond on 6-point Likert scale from 1 (strongly disagree) to 6 (strongly agree) how much they are aware of alternatives available to them ("I have many possible ways of behaving in any given situation"), their willingness and ability to be flexible and adapt to situations ("I am willing to work at creative solutions to problems"). Total scores range from 12 to 72, with higher scores indicating greater cognitive flexibility. The CFS has demonstrated good construct validity and internal consistency (Cronbach's α = .76 – .77). The internal consistency of the CFS in the current sample was acceptable (Cronbach's α = .77).
Engagement. Engagement with the program was assessed by recording whether participants attended the individual Be Well Plan sessions. Perceived session engagement was assessed after each session with a single item ("I felt engaged in the session."). Participants responded on a 5-point Likert scale from 1 (Strongly Disagree) to 5 (Strongly Agree).
Satisfaction. Participants' satisfaction with the program was assessed with a single item ("Overall, how satisfied were you with the program?") during the post-intervention assessment. Participants responded on a 5-point Likert scale from 1 (Very Dissatisfied) to 5 (Very Satisfied). Satisfaction with the individual Be Well Plan sessions was assessed after each session with a single item ("Overall, how satisfied were you with the session?"). Again, participants responded on a 5-point Likert scale from 1 (Very Dissatisfied) to 5

6a-i) Online questionnaires: describe if they were validated for online use and apply CHERRIES items to describe how the questionnaires were designed/deployed

If outcomes were obtained through online questionnaires, describe if they were validated for online use and apply CHERRIES items to describe how the questionnaires were designed/deployed [9].

	1	2	3	4	5	
subitem not at all important	○	○	○	○	○	essential

Does your paper address subitem 6a-i?

Copy and paste relevant sections from manuscript text

Meine Antwort

6a-ii) Describe whether and how "use" (including intensity of use/dosage) was defined/measured/monitored

Describe whether and how "use" (including intensity of use/dosage) was defined/measured/monitored (logins, logfile analysis, etc.). Use/adoption metrics are important process outcomes that should be reported in any ehealth trial.

	1	2	3	4	5	
subitem not at all important	○	○	○	○	○	essential

Does your paper address subitem 6a-ii?

Copy and paste relevant sections from manuscript text

Meine Antwort

6a-iii) Describe whether, how, and when qualitative feedback from participants was obtained

Describe whether, how, and when qualitative feedback from participants was obtained (e.g., through emails, feedback forms, interviews, focus groups).

	1	2	3	4	5	
subitem not at all important	○	○	○	○	○	essential

Does your paper address subitem 6a-iii?

Copy and paste relevant sections from manuscript text

Meine Antwort

6b) Any changes to trial outcomes after the trial commenced, with reasons

Does your paper address CONSORT subitem 6b? *

Copy and paste relevant sections from the manuscript (include quotes in quotation marks "like this" to indicate direct quotes from your manuscript), or elaborate on this item by providing additional information not in the ms, or briefly explain why the item is not applicable/relevant for your study

There were no changes after trial commencement; see also trial registration.

"The trial was ... registered with the Australian New Zealand Clinical Trial Registry (ACTRN12621000180819)."

7a) How sample size was determined

NPT: When applicable, details of whether and how the clustering by care provides or centers was addressed

7a-i) Describe whether and how expected attrition was taken into account when calculating the sample size

Describe whether and how expected attrition was taken into account when calculating the sample size.

	1	2	3	4	5	
subitem not at all important	○	○	○	○	○	essential

Does your paper address subitem 7a-i?

Copy and paste relevant sections from manuscript title (include quotes in quotation marks "like this" to indicate direct quotes from your manuscript), or elaborate on this item by providing additional information not in the ms, or briefly explain why the item is not applicable/relevant for your study

Meine Antwort

7b) When applicable, explanation of any interim analyses and stopping guidelines

Does your paper address CONSORT subitem 7b? *

Copy and paste relevant sections from the manuscript (include quotes in quotation marks "like this" to indicate direct quotes from your manuscript), or elaborate on this item by providing additional information not in the ms, or briefly explain why the item is not applicable/relevant for your study

No interim analyses were conducted; see also trial registration.

"The trial was ... registered with the Australian New Zealand Clinical Trial Registry (ACTRN12621000180819)."

8a) Method used to generate the random allocation sequence

NPT: When applicable, how care providers were allocated to each trial group

Does your paper address CONSORT subitem 8a? *

Copy and paste relevant sections from the manuscript (include quotes in quotation marks "like this" to indicate direct quotes from your manuscript), or elaborate on this item by providing additional information not in the ms, or briefly explain why the item is not applicable/relevant for your study

"After completing the baseline assessment, participants were randomised into either the intervention or wait-list control condition. As it was expected that some participants in the intervention group would not be able to commence the program due to unavailability, we chose a 2:1 allocation ratio for the intervention group. Randomisation was stratified by gender, performed by a researcher who was not involved in the delivery of the intervention using a random number generator (Randomness and Integrity Services Ltd, 2021)."

8b) Type of randomisation; details of any restriction (such as blocking and block size)

Does your paper address CONSORT subitem 8b? *

Copy and paste relevant sections from the manuscript (include quotes in quotation marks "like this" to indicate direct quotes from your manuscript), or elaborate on this item by providing additional information not in the ms, or briefly explain why the item is not applicable/relevant for your study

"After completing the baseline assessment, participants were randomised into either the intervention or wait-list control condition. As it was expected that some participants in the intervention group would not be able to commence the program due to unavailability, we chose a 2:1 allocation ratio for the intervention group. Randomisation was stratified by gender, performed by a researcher who was not involved in the delivery of the intervention using a random number generator (Randomness and Integrity Services Ltd, 2021)."

9) Mechanism used to implement the random allocation sequence (such as sequentially numbered containers), describing any steps taken to conceal the sequence until interventions were assigned

Does your paper address CONSORT subitem 9? *

Copy and paste relevant sections from the manuscript (include quotes in quotation marks "like this" to indicate direct quotes from your manuscript), or elaborate on this item by providing additional information not in the ms, or briefly explain why the item is not applicable/relevant for your study

"After completing the baseline assessment, participants were randomised into either the intervention or wait-list control condition. As it was expected that some participants in the intervention group would not be able to commence the program due to unavailability, we chose a 2:1 allocation ratio for the intervention group. Randomisation was stratified by gender, performed by a researcher who was not involved in the delivery of the intervention using a random number generator (Randomness and Integrity Services Ltd, 2021)."

"Participants from the wait-list control condition gained access to the program after the intervention group; facilitators were not aware whether they were delivering the program to the intervention or wait-list control condition."

10) Who generated the random allocation sequence, who enrolled participants, and who assigned participants to interventions

Does your paper address CONSORT subitem 10? *

Copy and paste relevant sections from the manuscript (include quotes in quotation marks "like this" to indicate direct quotes from your manuscript), or elaborate on this item by providing additional information not in the ms, or briefly explain why the item is not applicable/relevant for your study

"After completing the baseline assessment, participants were randomised into either the intervention or wait-list control condition. As it was expected that some participants in the intervention group would not be able to commence the program due to unavailability, we chose a 2:1 allocation ratio for the intervention group. Randomisation was stratified by gender, performed by a researcher who was not involved in the delivery of the intervention using a random number generator (Randomness and Integrity Services Ltd, 2021)."

11a) If done, who was blinded after assignment to interventions (for example, participants, care providers, those assessing outcomes) and how

NPT: Whether or not administering co-interventions were blinded to group assignment

11a-i) Specify who was blinded, and who wasn't

Specify who was blinded, and who wasn't. Usually, in web-based trials it is not possible to blind the participants [1, 3] (this should be clearly acknowledged), but it may be possible to blind outcome assessors, those doing data analysis or those administering co-interventions (if any).

	1	2	3	4	5	
subitem not at all important	○	○	○	○	○	essential

Does your paper address subitem 11a-i? *

Copy and paste relevant sections from the manuscript (include quotes in quotation marks "like this" to indicate direct quotes from your manuscript), or elaborate on this item by providing additional information not in the ms, or briefly explain why the item is not applicable/relevant for your study

"Participants from the wait-list control condition gained access to the program after the intervention group; facilitators were not aware whether they were delivering the program to the intervention or wait-list control condition."

11a-ii) Discuss e.g., whether participants knew which intervention was the "intervention of interest" and which one was the "comparator"

Informed consent procedures (4a-ii) can create biases and certain expectations - discuss e.g., whether participants knew which intervention was the "intervention of interest" and which one was the "comparator".

	1	2	3	4	5	
subitem not at all important	○	○	○	○	○	essential

Does your paper address subitem 11a-ii?

Copy and paste relevant sections from the manuscript (include quotes in quotation marks "like this" to indicate direct quotes from your manuscript), or elaborate on this item by providing additional information not in the ms, or briefly explain why the item is not applicable/relevant for your study

Meine Antwort

11b) If relevant, description of the similarity of interventions

(this item is usually not relevant for ehealth trials as it refers to similarity of a placebo or sham intervention to a active medication/intervention)

Does your paper address CONSORT subitem 11b? *

Copy and paste relevant sections from the manuscript (include quotes in quotation marks "like this" to indicate direct quotes from your manuscript), or elaborate on this item by providing additional information not in the ms, or briefly explain why the item is not applicable/relevant for your study

No active control condition was used in this trial:

"Participants in the wait-list control group were asked to complete the pre-and post-intervention assessment after which they were provided with access to the 5-week facilitated Be Well Plan sessions."

12a) Statistical methods used to compare groups for primary and secondary outcomes

NPT: When applicable, details of whether and how the clustering by care providers or centers was addressed

Does your paper address CONSORT subitem 12a? *

Copy and paste relevant sections from the manuscript (include quotes in quotation marks "like this" to indicate direct quotes from your manuscript), or elaborate on this item by providing additional information not in the ms, or briefly explain why the item is not applicable/relevant for your study

"Pre- and post-intervention differences were analysed with mixed ANOVAs in both Intention-to-treat (ITT; n = 75) and completer samples (n = 49)."

12a-i) Imputation techniques to deal with attrition / missing values

Imputation techniques to deal with attrition / missing values: Not all participants will use the intervention/comparator as intended and attrition is typically high in ehealth trials. Specify how participants who did not use the application or dropped out from the trial were treated in the statistical analysis (a complete case analysis is strongly discouraged, and simple imputation techniques such as LOCF may also be problematic [4]).

	1	2	3	4	5	
subitem not at all important	O	O	O	O	O	essential

Does your paper address subitem 12a-i? *

Copy and paste relevant sections from the manuscript (include quotes in quotation marks "like this" to indicate direct quotes from your manuscript), or elaborate on this item by providing additional information not in the ms, or briefly explain why the item is not applicable/relevant for your study

"Little's MCAR test was used to test whether data was missing completely at random, $\chi2(11) = 14.23$, $P = .22$, suggesting missing data in the seven outcomes measures was missing completely at random. Thus, for the ITT analyses we imputed missing data on the outcome measures for participants who did not complete the post-intervention assessment (n = 61, 37.2% of the analysed sample) using a Markov chain Monte Carlo method and information from the following variables: gender, age, ethnicity, working and student status, pre- and post-intervention scores in wellbeing, resilience, depression, anxiety, self-efficacy, sense of control and cognitive flexibility. In total, we simulated 10 new data sets using a maximum of 100 iterations from which mean scores for post-intervention outcomes were computed and used for the ITT analyses."

12b) Methods for additional analyses, such as subgroup analyses and adjusted analyses

Does your paper address CONSORT subitem 12b? *

Copy and paste relevant sections from the manuscript (include quotes in quotation marks "like this" to indicate direct quotes from your manuscript), or elaborate on this item by providing additional information not in the ms, or briefly explain why the item is not applicable/relevant for your study

"As there were small but significant age differences between the intervention and control conditions, we additionally performed separate mixed ANOVAs for all outcome measures while controlling for age in years. As age was a non-significant contributor in any of the analyses, the following results are presented without age as a covariate."

"Reliable change analysis was conducted by calculating a Reliable Change Index (RCI) using the method suggested by Jacobson and Truax (Jacobson & Truax, 1991). Separate RCIs for the ITT and the completer samples were calculated by subtracting participants' post-intervention scores from their baseline and subsequently dividing this difference score by the standard error of the difference for the measurements used."

X26) REB/IRB Approval and Ethical Considerations [recommended as subheading under "Methods"] (not a CONSORT item)

X26-i) Comment on ethics committee approval

	1	2	3	4	5	
subitem not at all important	○	○	○	○	○	essential

Does your paper address subitem X26-i?

Copy and paste relevant sections from the manuscript (include quotes in quotation marks "like this" to indicate direct quotes from your manuscript), or elaborate on this item by providing additional information not in the ms, or briefly explain why the item is not applicable/relevant for your study

Meine Antwort

x26-ii) Outline informed consent procedures

Outline informed consent procedures e.g., if consent was obtained offline or online (how? Checkbox, etc.?), and what information was provided (see 4a-ii). See [6] for some items to be included in informed consent documents.

	1	2	3	4	5	
subitem not at all important	○	○	○	○	○	essential

Does your paper address subitem X26-ii?

Copy and paste relevant sections from the manuscript (include quotes in quotation marks "like this" to indicate direct quotes from your manuscript), or elaborate on this item by providing additional information not in the ms, or briefly explain why the item is not applicable/relevant for your study

Meine Antwort

X26-iii) Safety and security procedures

Safety and security procedures, incl. privacy considerations, and any steps taken to reduce the likelihood or detection of harm (e.g., education and training, availability of a hotline)

	1	2	3	4	5	
subitem not at all important	○	○	○	○	○	essential

Does your paper address subitem X26-iii?

Copy and paste relevant sections from the manuscript (include quotes in quotation marks "like this" to indicate direct quotes from your manuscript), or elaborate on this item by providing additional information not in the ms, or briefly explain why the item is not applicable/relevant for your study

Meine Antwort

RESULTS

13a) For each group, the numbers of participants who were randomly assigned, received intended treatment, and were analysed for the primary outcome

NPT: The number of care providers or centers performing the intervention in each group and the number of patients treated by each care provider in each center

Does your paper address CONSORT subitem 13a? *

Copy and paste relevant sections from the manuscript (include quotes in quotation marks "like this" to indicate direct quotes from your manuscript), or elaborate on this item by providing additional information not in the ms, or briefly explain why the item is not applicable/relevant for your study

"Of the 126 participants who were allocated to the Be Well Plan condition, 51 (40.5%) participants did not commence the program. Participants not commencing the program reported other time commitments or unavailability for the scheduled session time (n = 44). There were no significant differences between participants from the intervention group who attended at least one Be Well Plan session (n = 75) and those who did not commence the program on any of the baseline outcome measures or demographic variables except for age; participants who did not commence with the program were on average 5.88 years younger, F(1, 125) = 9.81, P = .002."

13b) For each group, losses and exclusions after randomisation, together with reasons

Does your paper address CONSORT subitem 13b? (NOTE: Preferably, this is shown in a CONSORT flow diagram) *

Copy and paste relevant sections from the manuscript (include quotes in quotation marks "like this" to indicate direct quotes from your manuscript), or elaborate on this item by providing additional information not in the ms, or briefly explain why the item is not applicable/relevant for your study

From CONSORT flow diagram, Figure 1:

"Randomized to intervention (n=215)

Allocated to wait-list control (n=89):
- Lost to post-intervention assessment (n=35)
- Analysed in ITT analyses (n=89)
- Analysed in completer analyses (n=54)

Allocated to Be Well Plan (n=126):
- Did not start intervention (n=51)
- Lost to post-intervention assessment (n=26)
- Analysed in ITT analyses (n=75)
- Analysed in completer analyses (n=49)"

13b-i) Attrition diagram

Strongly recommended: An attrition diagram (e.g., proportion of participants still logging in or using the intervention/comparator in each group plotted over time, similar to a survival curve) or other figures or tables demonstrating usage/dose/engagement.

	1	2	3	4	5	
subitem not at all important	○	○	○	○	○	essential

Does your paper address subitem 13b-i?

Copy and paste relevant sections from the manuscript or cite the figure number if applicable (include quotes in quotation marks "like this" to indicate direct quotes from your manuscript), or elaborate on this item by providing additional information not in the ms, or briefly explain why the item is not applicable/relevant for your study

Meine Antwort

14a) Dates defining the periods of recruitment and follow-up

Does your paper address CONSORT subitem 14a? *

Copy and paste relevant sections from the manuscript (include quotes in quotation marks "like this" to indicate direct quotes from your manuscript), or elaborate on this item by providing additional information not in the ms, or briefly explain why the item is not applicable/relevant for your study

"University students were recruited between August 2020 and April 2021..."

"...in total ten individual groups were facilitated from August 2020 to June 2021."

14a-i) Indicate if critical “secular events” fell into the study period

Indicate if critical “secular events” fell into the study period, e.g., significant changes in Internet resources available or “changes in computer hardware or Internet delivery resources”

	1	2	3	4	5	
subitem not at all important	O	O	O	O	O	essential

Does your paper address subitem 14a-i?

Copy and paste relevant sections from the manuscript (include quotes in quotation marks "like this" to indicate direct quotes from your manuscript), or elaborate on this item by providing additional information not in the ms, or briefly explain why the item is not applicable/relevant for your study

Meine Antwort

14b) Why the trial ended or was stopped (early)

Does your paper address CONSORT subitem 14b? *

Copy and paste relevant sections from the manuscript (include quotes in quotation marks "like this" to indicate direct quotes from your manuscript), or elaborate on this item by providing additional information not in the ms, or briefly explain why the item is not applicable/relevant for your study

The trial did not end or was stopped early, see trial registration:

Trial Registration: Australian New Zealand Clinical Trial Registry ACTRN12621000180819.

15) A table showing baseline demographic and clinical characteristics for each group

NPT: When applicable, a description of care providers (case volume, qualification, expertise, etc.) and centers (volume) in each group

Does your paper address CONSORT subitem 15? *

Copy and paste relevant sections from the manuscript (include quotes in quotation marks "like this" to indicate direct quotes from your manuscript), or elaborate on this item by providing additional information not in the ms, or briefly explain why the item is not applicable/relevant for your study

"In the following, we will report demographic information of the 75 participants from the intervention group who attended at least one Be Well Plan session and the 89 participants who were allocated to the wait-list condition, see Table 2. On average, participants were 30.65 years old (SD = 10.10), with the majority being female (133/164, 81.1%), Caucasian (94/164, 57.9%; 48/164, 29.3% were Asian/Indian), and domestic students (122/164, 74.4%). The majority of participants were employed (part- or full-time, 87/164, 53.0%), while others did not work (42/164, 25.6%) or were unemployed/ had lost their job due to COVID-19 (25/164, 15.2%). On average, participants rated their overall health (M = 2.81, SD = 1.03) and their diet as fair/good (M = 2.78, SD = 1.06); their activity level as moderate (M = 1.76, SD = 0.72); and their sleep quality as fair/good (M = 7.35, SD = 2.78). Almost half of the sample (49.4%, n = 81) reported moderate to severe levels of depression, while over one-third (reported 36.0%, n = 59) reported moderate to severe levels of anxiety, suggesting the vulnerability of this student cohort."

15-i) Report demographics associated with digital divide issues

In ehealth trials it is particularly important to report demographics associated with digital divide issues, such as age, education, gender, social-economic status, computer/Internet/ehealth literacy of the participants, if known.

	1	2	3	4	5	
subitem not at all important	○	○	○	○	○	essential

Does your paper address subitem 15-i? *

Copy and paste relevant sections from the manuscript (include quotes in quotation marks "like this" to indicate direct quotes from your manuscript), or elaborate on this item by providing additional information not in the ms, or briefly explain why the item is not applicable/relevant for your study

"There were no significant differences between intervention and control groups on gender and ethnicity, nor were there differences on student or employment status or health status. However, there was a small but significant difference in age between the two conditions; participants allocated to the Be Well Plan intervention were on average 3.11 years older compared to participants in the wait-list control condition."

16) For each group, number of participants (denominator) included in each analysis and whether the analysis was by original assigned groups

16-i) Report multiple “denominators” and provide definitions

Report multiple “denominators” and provide definitions: Report N’s (and effect sizes) “across a range of study participation [and use] thresholds” [1], e.g., N exposed, N consented, N used more than x times, N used more than y weeks, N participants “used” the intervention/comparator at specific pre-defined time points of interest (in absolute and relative numbers per group). Always clearly define “use” of the intervention.

	1	2	3	4	5	
subitem not at all important	○	○	○	○	○	essential

Does your paper address subitem 16-i? *

Copy and paste relevant sections from the manuscript (include quotes in quotation marks "like this" to indicate direct quotes from your manuscript), or elaborate on this item by providing additional information not in the ms, or briefly explain why the item is not applicable/relevant for your study

From CONSORT flow diagram, Figure 1:

"Randomized to intervention (n=215)

Allocated to wait-list control (n=89):
- Lost to post-intervention assessment (n=35)
- Analysed in ITT analyses (n=89)
- Analysed in completer analyses (n=54)

Allocated to Be Well Plan (n=126):
- Did not start intervention (n=51)
- Lost to post-intervention assessment (n=26)
- Analysed in ITT analyses (n=75)
- Analysed in completer analyses (n=49)"

16-ii) Primary analysis should be intent-to-treat

Primary analysis should be intent-to-treat, secondary analyses could include comparing only "users", with the appropriate caveats that this is no longer a randomized sample (see 18-i).

	1	2	3	4	5	
subitem not at all important	○	○	○	○	○	essential

Does your paper address subitem 16-ii?

Copy and paste relevant sections from the manuscript (include quotes in quotation marks "like this" to indicate direct quotes from your manuscript), or elaborate on this item by providing additional information not in the ms, or briefly explain why the item is not applicable/relevant for your study

Meine Antwort

17a) For each primary and secondary outcome, results for each group, and the estimated effect size and its precision (such as 95% confidence interval)

Does your paper address CONSORT subitem 17a? *

Copy and paste relevant sections from the manuscript (include quotes in quotation marks "like this" to indicate direct quotes from your manuscript), or elaborate on this item by providing additional information not in the ms, or briefly explain why the item is not applicable/relevant for your study

"A detailed table outlining the scores for the Be Well Plan and wait-list control group for the primary and secondary outcomes, including effect sizes can be found in Table 3."

17a-i) Presentation of process outcomes such as metrics of use and intensity of use

In addition to primary/secondary (clinical) outcomes, the presentation of process outcomes such as metrics of use and intensity of use (dose, exposure) and their operational definitions is critical. This does not only refer to metrics of attrition (13-b) (often a binary variable), but also to more continuous exposure metrics such as "average session length". These must be accompanied by a technical description how a metric like a "session" is defined (e.g., timeout after idle time) [1] (report under item 6a).

	1	2	3	4	5	
subitem not at all important	O	O	O	O	O	essential

Does your paper address subitem 17a-i?

Copy and paste relevant sections from the manuscript (include quotes in quotation marks "like this" to indicate direct quotes from your manuscript), or elaborate on this item by providing additional information not in the ms, or briefly explain why the item is not applicable/relevant for your study

Meine Antwort

17b) For binary outcomes, presentation of both absolute and relative effect sizes is recommended

Does your paper address CONSORT subitem 17b? *

Copy and paste relevant sections from the manuscript (include quotes in quotation marks "like this" to indicate direct quotes from your manuscript), or elaborate on this item by providing additional information not in the ms, or briefly explain why the item is not applicable/relevant for your study

'Reliable change analysis showed that the majority (58/75, 77%) of participants demonstrated a significant reliable improvement in at least one of the primary outcomes. When looking at data for the completer sample, we found that a vast majority (40/49, 83%) of participants showed a reliable change in at least one outcome."

18) Results of any other analyses performed, including subgroup analyses and adjusted analyses, distinguishing pre-specified from exploratory

Does your paper address CONSORT subitem 18? *

Copy and paste relevant sections from the manuscript (include quotes in quotation marks "like this" to indicate direct quotes from your manuscript), or elaborate on this item by providing additional information not in the ms, or briefly explain why the item is not applicable/relevant for your study

"However, there was a small but significant difference in age between the two conditions; participants allocated to the Be Well Plan intervention were on average 3.11 years older compared to participants in the wait-list control condition."

"As age was a non-significant contributor in any of the analyses, the following results are presented without age as a covariate."

18-i) Subgroup analysis of comparing only users

A subgroup analysis of comparing only users is not uncommon in ehealth trials, but if done, it must be stressed that this is a self-selected sample and no longer an unbiased sample from a randomized trial (see 16-iii).

	1	2	3	4	5	
subitem not at all important	○	○	○	○	○	essential

Does your paper address subitem 18-i?

Copy and paste relevant sections from the manuscript (include quotes in quotation marks "like this" to indicate direct quotes from your manuscript), or elaborate on this item by providing additional information not in the ms, or briefly explain why the item is not applicable/relevant for your study

Meine Antwort

19) All important harms or unintended effects in each group

(for specific guidance see CONSORT for harms)

Does your paper address CONSORT subitem 19? *

Copy and paste relevant sections from the manuscript (include quotes in quotation marks "like this" to indicate direct quotes from your manuscript), or elaborate on this item by providing additional information not in the ms, or briefly explain why the item is not applicable/relevant for your study

"Only five participants (ITT: 5/75, 6.7%; completer: 5/49, 10.2%) reported a reliable deterioration in wellbeing, depression or anxiety: one participant showed a reliable decrease in wellbeing, whereas two participants showed reliable increases in depression or anxiety symptoms. Importantly, no participant showed reliable deteriorations in more than one of the mentioned outcomes. Furthermore, no participant reported any harmful effects from participation in the program."

19-i) Include privacy breaches, technical problems

Include privacy breaches, technical problems. This does not only include physical “harm” to participants, but also incidents such as perceived or real privacy breaches [1], technical problems, and other unexpected/unintended incidents. “Unintended effects” also includes unintended positive effects [2].

	1	2	3	4	5	
subitem not at all important	○	○	○	○	○	essential

Does your paper address subitem 19-i?

Copy and paste relevant sections from the manuscript (include quotes in quotation marks "like this" to indicate direct quotes from your manuscript), or elaborate on this item by providing additional information not in the ms, or briefly explain why the item is not applicable/relevant for your study

Meine Antwort

19-ii) Include qualitative feedback from participants or observations from staff/researchers

Include qualitative feedback from participants or observations from staff/researchers, if available, on strengths and shortcomings of the application, especially if they point to unintended/unexpected effects or uses. This includes (if available) reasons for why people did or did not use the application as intended by the developers.

	1	2	3	4	5	
subitem not at all important	○	○	○	○	○	essential

Does your paper address subitem 19-ii?

Copy and paste relevant sections from the manuscript (include quotes in quotation marks "like this" to indicate direct quotes from your manuscript), or elaborate on this item by providing additional information not in the ms, or briefly explain why the item is not applicable/relevant for your study

Meine Antwort

DISCUSSION

22) Interpretation consistent with results, balancing benefits and harms, and considering other relevant evidence

NPT: In addition, take into account the choice of the comparator, lack of or partial blinding, and unequal expertise of care providers or centers in each group

22-i) Restate study questions and summarize the answers suggested by the data, starting with primary outcomes and process outcomes (use)

Restate study questions and summarize the answers suggested by the data, starting with primary outcomes and process outcomes (use).

	1	2	3	4	5	
subitem not at all important	○	○	○	○	○	essential

Does your paper address subitem 22-i? *

Copy and paste relevant sections from the manuscript (include quotes in quotation marks "like this" to indicate direct quotes from your manuscript), or elaborate on this item by providing additional information not in the ms, or briefly explain why the item is not applicable/relevant for your study

"The current study examined the efficacy of a group-facilitated Internet-based program to promote mental health and wellbeing in a vulnerable population of university students. Compared to wait-list controls, participants in the intervention group significantly improved in all primary outcomes including mental wellbeing, resilience, depression, and anxiety as well as secondary outcomes including self-efficacy, sense of control and cognitive flexibility. Furthermore, participants' engagement and satisfaction with the Be Well Plan were examined showing that students were highly engaged and satisfied with

22-ii) Highlight unanswered new questions, suggest future research

Highlight unanswered new questions, suggest future research.

	1	2	3	4	5	
subitem not at all important	○	○	○	○	○	essential

Does your paper address subitem 22-ii?

Copy and paste relevant sections from the manuscript (include quotes in quotation marks "like this" to indicate direct quotes from your manuscript), or elaborate on this item by providing additional information not in the ms, or briefly explain why the item is not applicable/relevant for your study

Meine Antwort

20) Trial limitations, addressing sources of potential bias, imprecision, and, if relevant, multiplicity of analyses

20-i) Typical limitations in ehealth trials

Typical limitations in ehealth trials: Participants in ehealth trials are rarely blinded. Ehealth trials often look at a multiplicity of outcomes, increasing risk for a Type I error. Discuss biases due to non-use of the intervention/usability issues, biases through informed consent procedures, unexpected events.

	1	2	3	4	5	
subitem not at all important	○	○	○	○	○	essential

Does your paper address subitem 20-i? *

Copy and paste relevant sections from the manuscript (include quotes in quotation marks "like this" to indicate direct quotes from your manuscript), or elaborate on this item by providing additional information not in the ms, or briefly explain why the item is not applicable/relevant for your study

"While there were several strengths of the study, several limitations need to be discussed. First, the study used a wait-list control group as comparison; while, wait-list groups are cost-effective and ethical alternative control conditions, they might exaggerate effects sizes compared to other control conditions (e.g., no intervention or active psychological placebo conditions) (Furukawa et al., 2014). Future studies should test the Be Well Plan against an active, psychological placebo control group. Second, while the study population (i.e., university students) was a vulnerable group, it does limit the generalisability of the findings to the general public. For instance, the general public typically reports better mental health, which in turn means that effect sizes in the general public may be considerably lower. Digital literacy may also be higher in a student population, potentially affecting accessibility and scalability for a broader range of specific or general community groups. Future work should be conducted to further test the Be Well Plan in different population cohorts. Third, the study was not sufficiently powered to find significant effects for depression and anxiety in the completer analysis, despite medium effect sizes. While almost half of participants showed reliable improvements in depression and anxiety outcomes, future studies should include larger samples to allow to test for small to medium effects sies in psychological distress with clinical samples. Fourth, there was a high number of participants who registered their interest, completed the baseline measures and were randomized to the intervention group but did not commence the first session. While a limitation, this is common with Internet-based programs; for example, a variety of studies in the area have noted similar engagement (uptake and adherence) challenges (Ali, Fassnacht, et al., 2022; Batterham & Calear, 2017; Musiat et al., 2014). Fifth, the current findings are short-term results only as our post assessment was taken at one week post final session. Literature clearly indicates that the impact of mental health interventions diminishes over time, particularly in general wellbeing programs. While investigating the long-term impact might look like an interesting question, the literature on diminishing returns is well established (van Agteren, Iasiello, Lo, et al., 2021a). Rather, it is arguably more important to invest effort in designing and testing sustainable booster material (Andersson et al., 2014). Development work is currently underway to develop ongoing topical booster sessions, which aim to both reinforce core program learnings and introduce new content and activities over time. Finally, most participants were female. While males who did participate in the program, benefitted equally as female participants, there is an urgent need to attract and engage more males in programs such as the Be Well

21) Generalisability (external validity, applicability) of the trial findings

NPT: External validity of the trial findings according to the intervention, comparators, patients, and care providers or centers involved in the trial

21-i) Generalizability to other populations

Generalizability to other populations: In particular, discuss generalizability to a general Internet population, outside of a RCT setting, and general patient population, including applicability of the study results for other organizations

	1	2	3	4	5	
subitem not at all important	○	○	○	○	○	essential

Does your paper address subitem 21-i?

Copy and paste relevant sections from the manuscript (include quotes in quotation marks "like this" to indicate direct quotes from your manuscript), or elaborate on this item by providing additional information not in the ms, or briefly explain why the item is not applicable/relevant for your study

Meine Antwort

21-ii) Discuss if there were elements in the RCT that would be different in a routine application setting

Discuss if there were elements in the RCT that would be different in a routine application setting (e.g., prompts/reminders, more human involvement, training sessions or other co-interventions) and what impact the omission of these elements could have on use, adoption, or outcomes if the intervention is applied outside of a RCT setting.

	1	2	3	4	5	
subitem not at all important	○	○	○	○	○	essential

Does your paper address subitem 21-ii?

Copy and paste relevant sections from the manuscript (include quotes in quotation marks "like this" to indicate direct quotes from your manuscript), or elaborate on this item by providing additional information not in the ms, or briefly explain why the item is not applicable/relevant for your study

Meine Antwort

OTHER INFORMATION

23) Registration number and name of trial registry

Does your paper address CONSORT subitem 23? *

Copy and paste relevant sections from the manuscript (include quotes in quotation marks "like this" to indicate direct quotes from your manuscript), or elaborate on this item by providing additional information not in the ms, or briefly explain why the item is not applicable/relevant for your study

Trial Registration: Australian New Zealand Clinical Trial Registry ACTRN12621000180819

24) Where the full trial protocol can be accessed, if available

Does your paper address CONSORT subitem 24? *

Cite a Multimedia Appendix, other reference, or copy and paste relevant sections from the manuscript (include quotes in quotation marks "like this" to indicate direct quotes from your manuscript), or elaborate on this item by providing additional information not in the ms, or briefly explain why the item is not applicable/relevant for your study

No full trial protocol was published.

25) Sources of funding and other support (such as supply of drugs), role of funders

Does your paper address CONSORT subitem 25? *

Copy and paste relevant sections from the manuscript (include quotes in quotation marks "like this" to indicate direct quotes from your manuscript), or elaborate on this item by providing additional information not in the ms, or briefly explain why the item is not applicable/relevant for your study

"The delivery of the Be Well Plan was supported with funding from the College of Education, Psychology and Social Work, Flinders University, Adelaide, Australia."

X27) Conflicts of Interest (not a CONSORT item)

X27-i) State the relation of the study team towards the system being evaluated

In addition to the usual declaration of interests (financial or otherwise), also state the relation of the study team towards the system being evaluated, i.e., state if the authors/evaluators are distinct from or identical with the developers/sponsors of the intervention.

	1	2	3	4	5	
subitem not at all important	○	○	○	○	○	essential

Does your paper address subitem X27-i?

Copy and paste relevant sections from the manuscript (include quotes in quotation marks "like this" to indicate direct quotes from your manuscript), or elaborate on this item by providing additional information not in the ms, or briefly explain why the item is not applicable/relevant for your study

Meine Antwort

About the CONSORT EHEALTH checklist

As a result of using this checklist, did you make changes in your manuscript? *

- ○ yes, major changes
- ◉ yes, minor changes
- ○ no

What were the most important changes you made as a result of using this checklist?

Meine Antwort

How much time did you spend on going through the checklist INCLUDING making changes in your manuscript *

Approx. 2-3 hours were spent on completing the checklist.

As a result of using this checklist, do you think your manuscript has improved? *

- [x] yes
- [ ] no
- [ ] Sonstiges:

Would you like to become involved in the CONSORT EHEALTH group?

This would involve for example becoming involved in participating in a workshop and writing an "Explanation and Elaboration" document

- [ ] yes
- [ ] no
- [ ] Sonstiges:

Any other comments or questions on CONSORT EHEALTH

Meine Antwort

STOP - Save this form as PDF before you click submit

To generate a record that you filled in this form, we recommend to generate a PDF of this page (on a Mac, simply select "print" and then select "print as PDF") before you submit it.

When you submit your (revised) paper to JMIR, please upload the PDF as supplementary file.

Don't worry if some text in the textboxes is cut off, as we still have the complete information in our database. Thank you!

Final step: Click submit !

Click submit so we have your answers in our database!

Senden

Alle Eingaben löschen

Geben Sie niemals Passwörter über Google Formulare weiter.

Dieser Inhalt wurde nicht von Google erstellt und wird von Google auch nicht unterstützt. Missbrauch melden - Nutzungsbedingungen - Datenschutzerklärung

Google Formulare
